# Decelerated dinosaur skull evolution with the origin of birds

**DOI:** 10.1371/journal.pbio.3000801

**Published:** 2020-08-18

**Authors:** Ryan N. Felice, Akinobu Watanabe, Andrew R. Cuff, Michael Hanson, Bhart-Anjan S. Bhullar, Emily R. Rayfield, Lawrence M. Witmer, Mark A. Norell, Anjali Goswami

**Affiliations:** 1 Centre for Integrative Anatomy, Department of Cell and Developmental Biology, University College London, London, United Kingdom; 2 Life Sciences Department, Vertebrates Division, Natural History Museum, London, United Kingdom; 3 Department of Anatomy, New York Institute of Technology College of Osteopathic Medicine, Old Westbury, New York, United States of America; 4 Division of Paleontology, American Museum of Natural History, New York, New York, United States of America; 5 Hull York Medical School, University of York, York, United Kingdom; 6 Department of Earth & Planetary Sciences, Yale University, New Haven, Connecticut, United States of America; 7 Yale Peabody Museum of Natural History, Yale University, New Haven, Connecticut, United States of America; 8 School of Earth Sciences, University of Bristol, Bristol, United Kingdom; 9 Department of Biomedical Sciences, Ohio University Heritage College of Osteopathic Medicine, Athens, Ohio, United States of America; Ecole Normale Supérieure, FRANCE

## Abstract

The evolutionary radiation of birds has produced incredible morphological variation, including a huge range of skull form and function. Investigating how this variation arose with respect to non-avian dinosaurs is key to understanding how birds achieved their remarkable success after the Cretaceous–Paleogene extinction event. Using a high-dimensional geometric morphometric approach, we quantified the shape of the skull in unprecedented detail across 354 extant and 37 extinct avian and non-avian dinosaurs. Comparative analyses reveal fundamental differences in how skull shape evolved in birds and non-avian dinosaurs. We find that the overall skull shape evolved faster in non-avian dinosaurs than in birds across all regions of the cranium. In birds, the anterior rostrum is the most rapidly evolving skull region, whereas more posterior regions—such as the parietal, squamosal, and quadrate—exhibited high rates in non-avian dinosaurs. These fast-evolving elements in dinosaurs are strongly associated with feeding biomechanics, forming the jaw joint and supporting the jaw adductor muscles. Rapid pulses of skull evolution coincide with changes to food acquisition strategies and diets, as well as the proliferation of bony skull ornaments. In contrast to the appendicular skeleton, which has been shown to evolve more rapidly in birds, avian cranial morphology is characterised by a striking deceleration in morphological evolution relative to non-avian dinosaurs. These results may be due to the reorganisation of skull structure in birds—including loss of a separate postorbital bone in adults and the emergence of new trade-offs with development and neurosensory demands. Taken together, the remarkable cranial shape diversity in birds was not a product of accelerated evolution from their non-avian relatives, despite their frequent portrayal as an icon of adaptive radiations.

## Abstract 2

Using high-density morphometrics to quantify skull shape in birds and non-avian dinosaurs, this study reveals that the skull evolves at significantly slower rate in birds compared to other dinosaurs. The macroevolutionary history of the dinosaur skull reflects the mosaic functional, structural, and developmental changes in the skull across the origin of birds.

## Introduction

Among tetrapods, extant birds exhibit incredible taxonomic and ecomorphological diversity, comprising over 10,000 extant species that occupy myriad niches on 7 continents [1–3]. They possess a number of specialised traits that have been proposed as key innovations that facilitated their radiation, including the keeled pectoral girdle and flight stroke [4,5]; a hindlimb capable of perching [6]; a short, fused caudal axial skeleton [7]; an air-sac–based respiratory system [8]; an edentulous beak [9]; and highly encephalised brains [10,11] among other traits. Did this broad suite of phenotypic changes result in enhanced disparity and rates of phenotypic evolution in birds compared to their non-avian relatives? The rich fossil record shows that the stem group of birds, the non-avian dinosaurs, also exhibit remarkable variation in body plan, body size, and trophic ecology [12,13]. As such, understanding the context of avian diversification requires an in-depth investigation of the dynamics of tempo and mode of phenotypic evolution across Dinosauria as a whole [9,14–21].

Previous research comparing macroevolutionary patterns between avian and non-avian dinosaurs has focused on the post-cranial skeleton and/or body size [16–18,20,22–24]. Many of these studies have shown significant heterogeneity in disparity and evolutionary rates across dinosaur clades and across regions of the body. However, there remains some debate whether the radiation of crown birds represents a shift in tempo and mode of evolution or a continuation of existing trends. For example, some studies quantifying rates of phenotypic evolution across the origin of birds have recovered sustained high evolutionary rates across the entire avian stem lineage [18], while others have suggested that birds indeed evolve under different rates or adaptive regimes compared to non-avian dinosaurs [16,24]. Still others have found an isolated acceleration of evolutionary rate before the origin of birds, at the root of Paraves [17]. Despite this uncertainty about the timing of rate shifts in dinosaur macroevolution, there is an emerging consensus that there is heterogeneity in the tempo and mode of evolution across body regions [15,20] and across lineages [16–18,22].

Fewer studies have focused on the tempo and mode of craniofacial evolution specifically, despite many of the key ‘avian’ features being localised to the skull (e.g., edentulous beak, kinetic palate, encephalised brain). These studies have primarily been limited to the use of linear or two-dimensional (2D) morphometric data. These analyses support the idea that avian cranial diversity is aligned along a similar axis of variation as in non-avian dinosaurs, with similar trends in braincase to face proportions [25]. However, these efforts have largely been restricted to Theropoda [21,26–28], excluding much of total non-avian dinosaur diversity.

Analysis of avian cranial skeletal evolution using three-dimensional (3D) morphometric data has revealed a mosaic pattern of evolution [29]. The components of the skull evolve at different tempo and mode, with high rate variability among lineages [29,30]. Rate and disparity are positively correlated, with the anterior face evolving fastest and achieving the highest disparity. In contrast, the posterior and ventral braincase evolves relatively slowly and has low variance. This heterogeneity in rate and disparity across skull regions supports the hypothesis that the mosaic assembly of the avian skull has contributed to the vast phenotypic and ecological disparity within the clade [29,31]. Birds and non-avian dinosaurs exhibit some similarities in cranial integration patterns, further suggesting that avian cranial variation forms a continuum with other members of Dinosauria. For example, the regions that are strongly integrated in birds, such as the occiput, are also highly integrated in non-avian dinosaurs [32]. However, it is currently unknown whether these common integration patterns across Dinosauria reflect similar patterns of mosaicism in avian and non-avian dinosaurs.

Here, we test whether the origin of birds is marked with a distinct shift in cranial evolutionary dynamics. Specifically, we assess how the macroevolutionary patterns generating avian cranial diversity compares to those giving rise to the diversity of cranial morphology across Dinosauria as a whole. We address 2 main questions. First, did the same regions of the skull evolve at high rates and exhibit high disparity in non-avian dinosaurs as in birds? We hypothesise that because of the major differences in cranial anatomy, function, development, and phenotypic integration, non-avian and avian dinosaurs exhibit distinct patterns of regionalisation of variability within the skull. Second, do birds and non-avian dinosaurs evolve at different rates? Previous studies quantifying cranial form across dinosaurs and birds have relied on limited length measurements or a small set of 2D landmarks because of limited points of clear homology [21,26–28]. To comprehensively represent the complex morphology and diversity of the dinosaur skull, we used a high-dimensional 3D geometric morphometric approach to quantify shape morphology across the entire surface of the skull in both extant birds (*n* = 354) and non-avialan dinosaurs (*n* = 36), as well as the Cretaceous ornithuran bird *Hesperornis*. Using semiautomated placement of surface semi-landmarks [33], we projected a total of 775 surface semi-landmarks onto each specimen (S1 Fig), allowing for the disparate morphology of non-avian and avian dinosaurs to be compared in a single unified Procrustes space [33]. Because this study concerns 3D shape of the entire skull, we selected only highly complete, articulated, and undeformed fossil specimens. We also included several specimens that were digitally reconstructed, restored, or retrodeformed [34–37]. Although these requirements reduced the total number of extinct taxa in the dataset, it also maximised the quality of the data and minimised missing data. This dataset contains representatives of all major groups, including 4 sauropodomorphs, 4 thyreophorans, 4 hadrosaurs, 3 pachycephalosaurs, 5 ceratopsians, and 15 non-avian theropods S2 Table). Although the total sampling in this study represents only a fraction of the overall diversity of dinosaurs, the sampling is proportionate for avian and non-avian dinosaurs, representing approximately 3% of known species. We sample over 80% of extant avian families, with non-avian sampling covering most major clades and the breadth of cranial variation. Moreover, the total number of specimens in the present analysis is similar to or exceeds the taxonomic sampling of other analyses of cranial shape in dinosaurs or birds, all of which use a much less detailed characterisation of morphology with 2D geometric morphometrics (e.g., *n* = 22 [21], *n* = 36 [27], *n* = 41 [28], *n* = 108 [26]). We additionally carried out post hoc analyses, detailed below, to test for statistical artefacts related to sampling.

Because of the variable completeness of the fossil specimens, we conducted all analyses concerning rate and disparity of a region (e.g., a whole bone or phenotypic module composed of multiple bones) using all specimens that preserve that region or element. For analyses concerning rate and disparity across the entire skull, we performed tests using 2 subsets of the total sample: one consisting of those taxa that preserve the dorsal and lateral sides of the skull (*n* = 28) and composed of 9 anatomical modules (S2 Table) and the other consisting of those taxa preserving the occiput and quadrate as well (11 anatomical modules, *n* = 19). We then used modern comparative methods to estimate phenotypic rates of evolution across time, taxa, and cranial regions.

## Results

**Fig 1:**
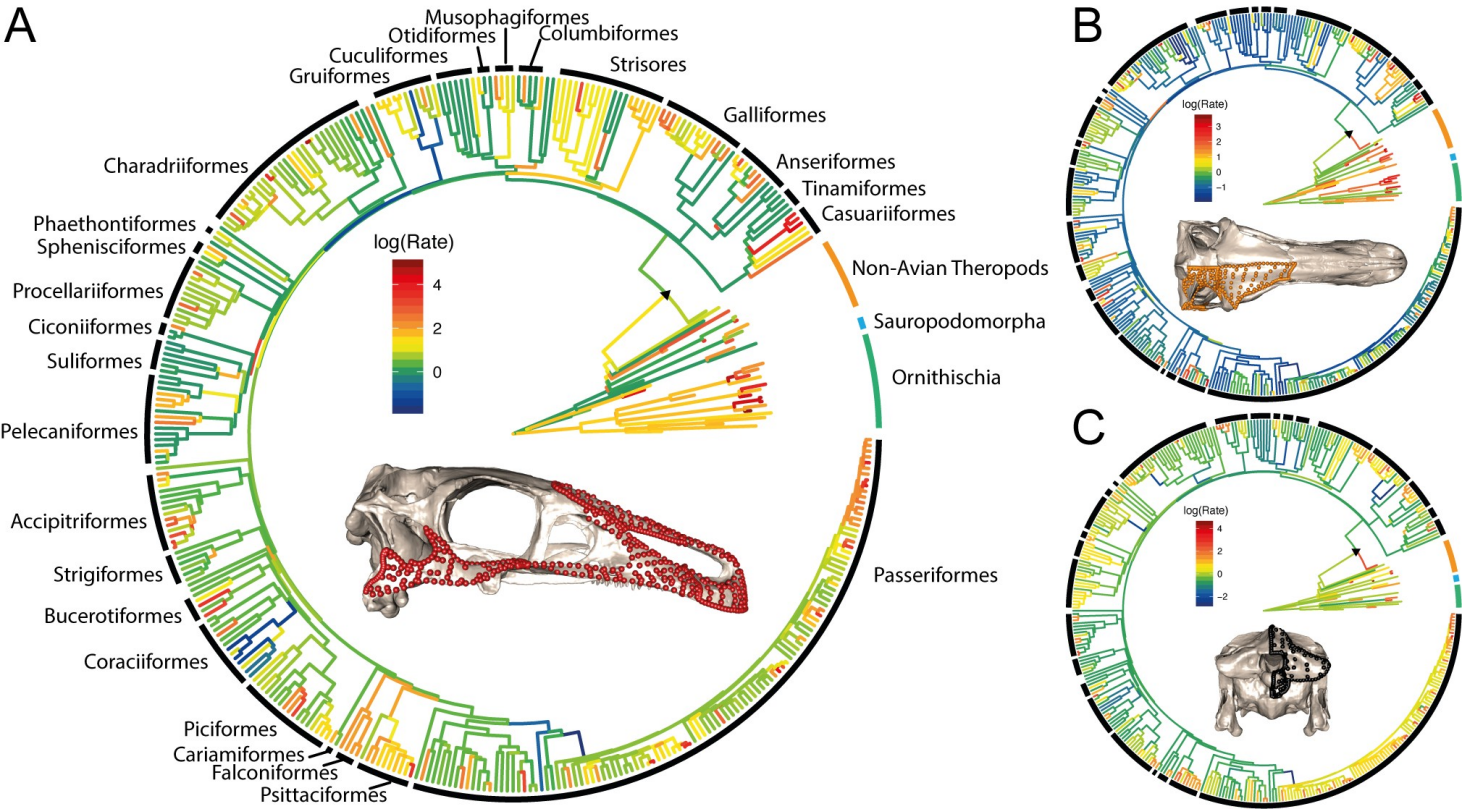
Estimation of rates of cranial evolution on a time-calibrated phylogeny of Dinosauria using a variable-rates BM model of evolution. (A) In the rostrum, ornithischians evolved faster than avian and non-avian theropods. (B) The cranial vault evolves fastest in non-avian dinosaurs with bony cranial ornaments. (C) Rates of evolution are generally conserved and low in the occipital region, with slightly elevated rates in Passeriformes and in pachycephalosaurs. Black triangle indicates the origin of Aves. See S2–S36 Figs for estimated rates for all cranial regions and phylogenetic hypotheses and detailed tip labels. Data and code archived at www.github.com/rnfelice/Dinosaur_Skulls. BM, Brownian Motion.

**Fig 2:**
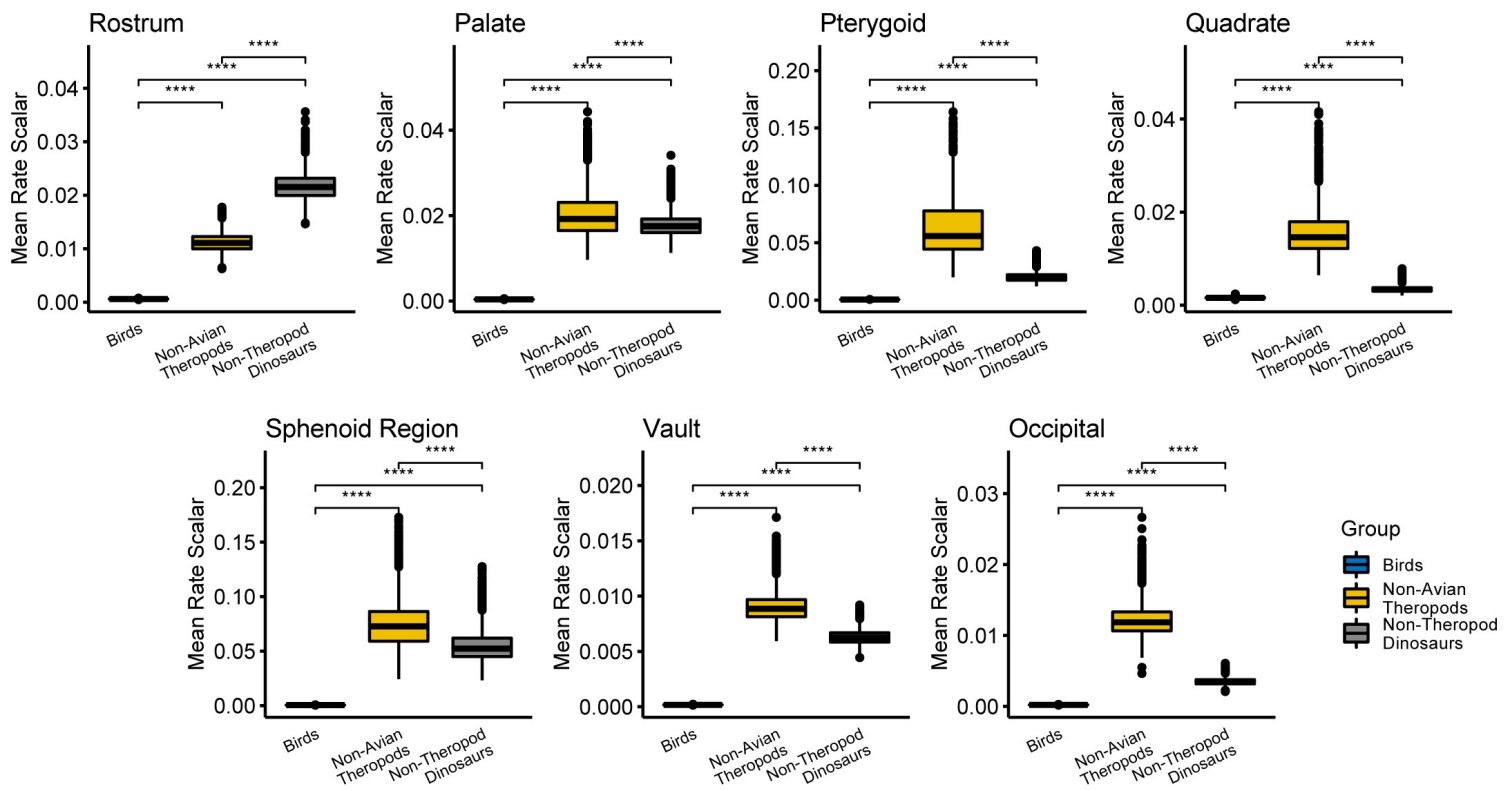
Comparison of per-group evolutionary rate scalars. Birds do not have the highest rates of evolution in any cranial region. For each group, mean rate scalar is the mean of the rate scalars in the post-burn-in posterior distribution under the variable-rates evolutionary model estimated using BayesTraits under the traditional Dinosaur phylogenetic hypothesis (Saurischia and Ornithischia as sister clades). Mean rates were scaled to the sum of the branch lengths in the corresponding subtree. Mean rate scalars were compared between groups using non-parametric *t* tests; significantly different distributions are indicated with ****p < 0.00005. These results are robust to subsampling of taxa (S1 Fig 37), Procrustes superimposition (S1 Fig 38) and alternative methods for comparing evolutionary rates (S39–S42 Figs). Data and code archived at www.github.com/rnfelice/Dinosaur_Skulls.

Under a variable-rates Brownian Motion (BM) model [38], all cranial regions evolved more slowly in birds than in either non-avian theropods, non-theropod dinosaurs (sauropodomorphs and ornithischians), or both (Fig 1 and S2–S22 Figs). The distribution of rates is highly variable across cranial regions and across taxa, but the highest rates are consistently observed on branches within non-avian dinosaurs rather than those within birds. These results are robust to the choice of phylogenetic topology and tree dating approach. Comparing per-branch evolutionary rate scalars demonstrates that birds evolved significantly more slowly than non-avian theropods and non-theropod dinosaurs in all cranial regions (Fig 2). These results are robust to taxonomic subsampling (S37 Fig) and changes in Procrustes alignment (S38 Fig) and are further supported by rate comparisons using the multivariate K (σ_mult_) rate statistic [39] that assumes equal-rates BM (S39–S42 Figs, S1 Table)

For each skull region, variable-rates evolutionary models illustrate which subclades are driving the differences in rates between non-avian dinosaurs and birds (Fig 1). In the rostrum (premaxilla, maxilla, nasal, jugal), rapid evolution is particularly evident in *Diplodocus* and *Lambeosaurus*, which convergently have distinct cranial configurations with posterodorsally positioned nasals and elongate premaxillae compared to their closest relatives in this analysis (Fig 1A and S2–S4 Figs). Among non-avian theropods, the oviraptorosaurs *Citipati* and *Incisivosaurus* have relatively high rates of rostrum evolution. Although they represent sister taxa in our sampling, these 2 genera exhibit widely divergent cranial morphotypes (e.g., *Citipati* has an edentulous premaxilla, unlike *Incisivosaurus*). Along the early avialan lineages (including *Hesperornis*), rates of rostrum evolution are slightly higher, but the rates along the branch leading to crown Aves and basal branches within the crown group are relatively slow compared to non-avian dinosaurs.

Within non-avian dinosaurs, evolutionary pulses in the cranial vault are associated with cranial ornaments. Ornithischian dinosaurs, such as horned and crested ceratopsians and dome-headed pachycephalosaurs, exhibit elevated rates for this region (Fig 1B and S5–S7 Figs). Similarly, high rates are seen within non-avian Theropoda, where parietal crests are present in taxa such as *Tyrannosaurus* and *Allosaurus*, although these structures are related to muscle attachment rather than display [40]. These bony elaborations of the skull roof are relatively rare in birds [41]. However, per-bone analyses limited to non-avian dinosaurs indicate that rate heterogeneity in the vault is driven not only by ornamentation. Indeed, several unornamented theropods also exhibit high rates of vault evolution on terminal branches, including those leading to *Ornithomimus*, *Struthiomimus*, and *Incisivosaurus* (S5–S7 Figs). In the frontal, non-avian theropods underwent faster evolution compared to ornithischians (S23 Fig), although the ceratopsids *Triceratops*, *Chasmosaurus*, and *Diabloceratops* also exhibit high evolutionary rates in the frontal, presumably reflecting the reorganisation of the skull roof to support the postorbital horns present in these species. In contrast, the parietal evolved faster in ornithischians than in saurischians (S24 Fig). The slowest-evolving ornithischians are relatively unornamented (e.g., *Stegosaurus*, *Thescelosaurus*, and *Pinacosaurus*), and theropods with fast-evolving parietals are tyrannosaurids with parietal (nuchal) crests (e.g., *Tyrannosaurus*, *Teratophoneus*, and *Alioramus*). As with the parietal, the squamosal generally evolved fastest in ornithischians and slowly in saurischians (S25 Fig).

**Fig 3:**
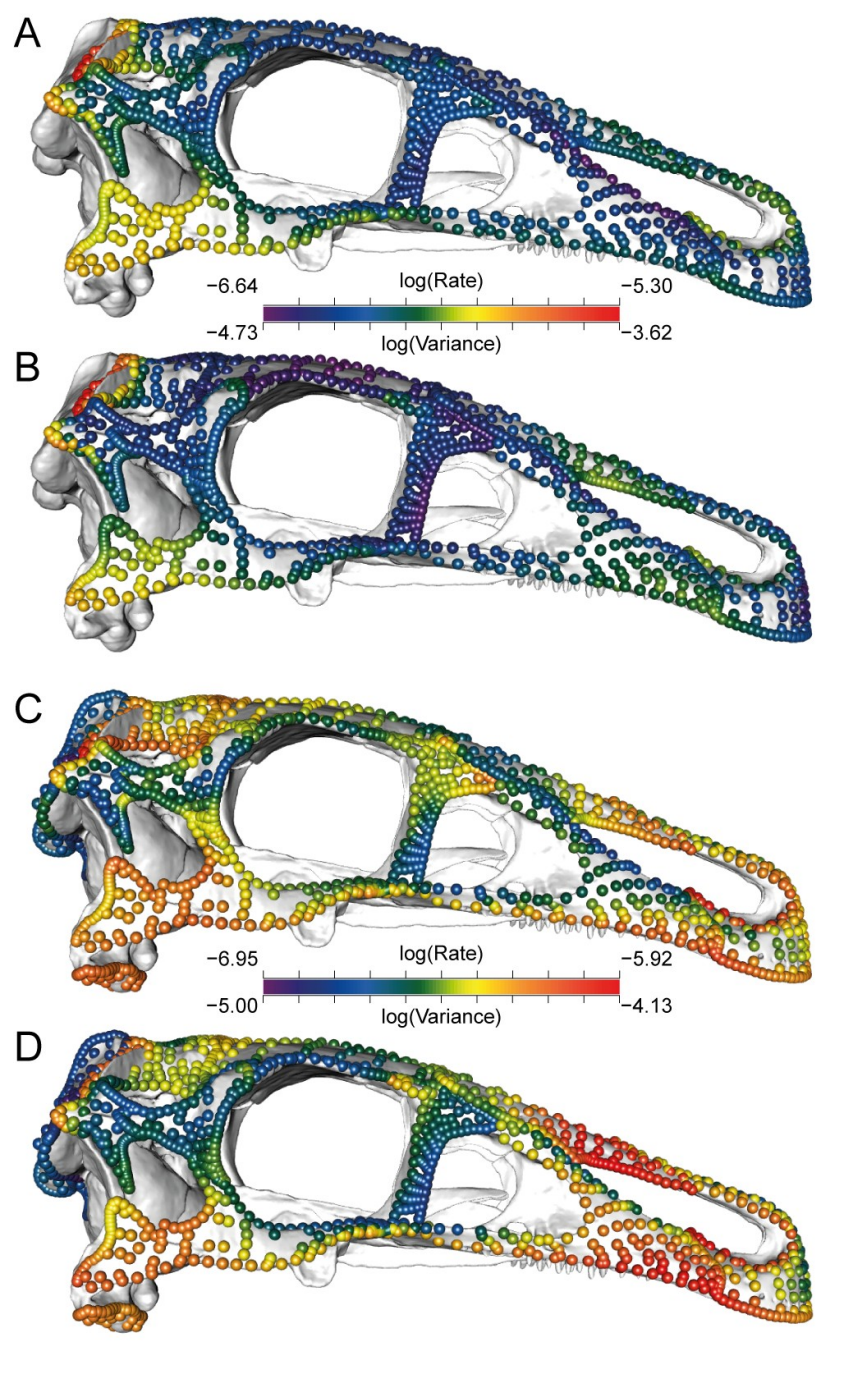
Per-landmark evolutionary rates (under single-rate BM model) and Procrustes variance. Landmarks and sliding semi-landmarks represented in the 9-module dataset (A, B) and 11-module dataset (C, D), illustrated on the skull of *Erlikosaurus andrewsi* (IGM 100/111). Landmarks are coloured according to evolutionary rate (A, C) and Procrustes variance (B, D), where a warmer colour indicates greater value. Data and code archived at www.github.com/rnfelice/Dinosaur_Skulls. BM, Brownian Motion; IGM, Paleontological Center, Mongolian Academy of Sciences.

The quadrate and pterygoid form a strongly integrated unit with relatively slow evolutionary rates in crown birds [29]. In contrast, the quadrate exhibits high rates across non-avian dinosaurs in the 11-module analyses (Fig 3C). However, when analysed separately from other skull elements, such that only shape—rather than both shape and relative position—is represented, the quadrate exhibits apparently low evolutionary rates in non-avian dinosaurs than in crown birds (S14–S16 Figs). Indeed, quadrate shape evolution is slow among non-avian dinosaurs, except in ornithomimosaurs and *Plateosaurus* (S14–S16 Figs). This difference reflects the effects of Procrustes alignment with other components of the skull, which preserves information about relative positions of elements, compared to separate Procrustes alignments for each element that characterises only the shape of the region of interest. When σ_mult_ rate comparison tests are carried out on the quadrate landmarks from the global Procrustes alignment, non-avian dinosaurs exhibit significantly faster evolution than birds (S42 Fig). The aspect of the quadrate that evolves rapidly in non-avian dinosaurs, therefore, seems to be the relative position of the articular surface of the quadrate relative to the rest of the skull, rather than its shape. In contrast, the pterygoid shows a high rate of evolution along the branch leading to Avialae (S17–S19 Figs). This suggests that the derived, strut-like pterygoid of birds and concomitant loss of the ectopterygoid evolved rapidly immediately preceding the origin of the crown group. Among non-avian dinosaurs, there is an increase in evolutionary rate of the pterygoid at the origin of each of the major clades (Theropoda, Sauropodomorpha, and Ornithischia) and Ornithomimosauria.

The occiput shows slow rates of evolution in both birds [29] and non-avian dinosaurs (S8–S10 Figs), a feature that may be conserved across tetrapods [42,43]. Nonetheless, ornithomimosaurs show bursts of rapid evolution in the occiput, driving its evolution to be higher in non-avian theropods than crown birds (Figs 1C and 2, S8–S10 Figs). High rates of occipital evolution are also present in pachycephalosaurs, reflecting their derived occipital morphology which has been interpreted as supporting increased epaxial cervical musculature, hypothesised as an adaptation for their inferred headbutting behaviour [44,45].

Examining patterns of disparity and evolutionary rates across the skull demonstrates major differences in how the skull evolved in in non-avian and avian dinosaurs. Non-avian dinosaurs show high rates of evolution predominantly in the posterodorsal parts of the skull. This pattern contrasts with birds, which have the highest rates of evolution in the anterior-most parts of the face [46]. In the 9-module dataset, per-landmark evolutionary rates show that the fastest evolving region—the parietal (S43 Fig, σ_mult_ = 4.53 × 10^−7^)—evolves approximately 2.7 times faster than the slowest-evolving modules: the frontal and the lacrimal (frontal: σ_mult_ = 1.64 × 10^−7^; lacrimal: σ_mult_ = 1.69 × 10^−7^). This difference reflects the inclusion of a larger sample of ceratopsians in that dataset, which possess elaborate frills formed by the parietal and squamosal (Fig 3A). The squamosal and the quadratojugal show moderately high rates, and the remainder of the skull evolves relatively slowly (Fig 3A).

Skull shape disparity, as calculated by Procrustes variance, exhibits similar patterns to rates across the skull, where regions that evolved rapidly are also the most disparate. The fast-evolving parietal and quadratojugal exhibit the highest variance, whereas the frontal and lacrimal + prefrontal have the lowest variance across avian and non-avian dinosaurs (Fig 3). In all regions except the quadrate, birds have significantly lower disparity than non-avian dinosaur groups (S44 Fig, S2 Table and S3 Table), which is congruent with the pattern observed with evolutionary rates.

In the 11-module dataset with fewer specimens represented (notably, excluding ceratopsians) the parietal and quadratojugal retain high evolutionary rates (Fig 3C and S43 Fig), as observed in the 9-module dataset. The other parts of the skull exhibit greater variation in rates compared to the 9-module dataset. The quadrate underwent the highest rate of evolution, and the frontal, nasal, premaxilla, palatal surface of the maxilla, and prefrontal also evolved rapidly. In contrast, the occiput, postorbital, squamosal, ventral lacrimal, and dorsal maxilla evolved slowly (Fig 3C and S43 Fig).

**Fig 4:**
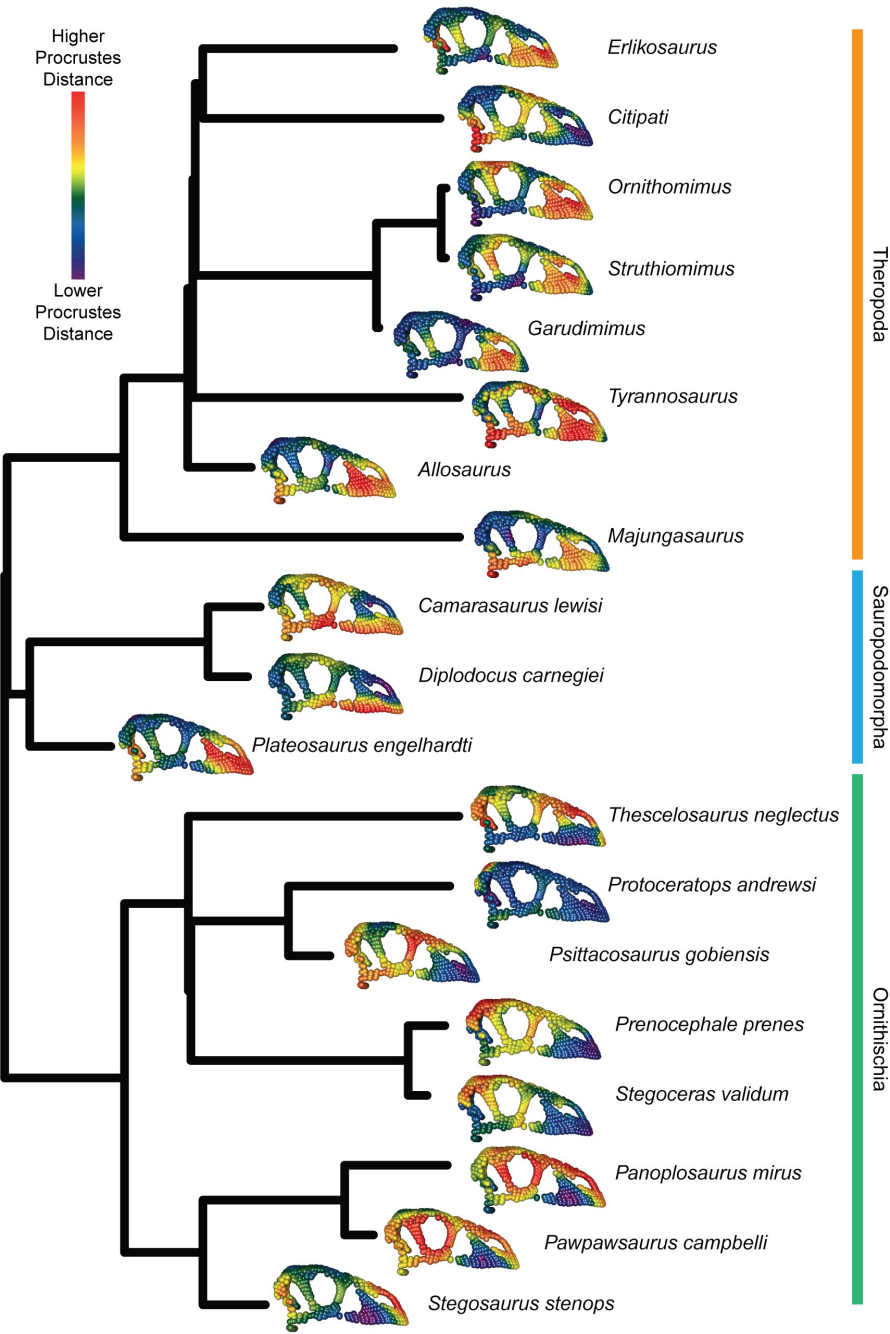
Phenotypic difference between each specimen for each landmark in the 11-module dataset and the mean skull shape. For each specimen, the mean landmark configuration is plotted with points coloured relative to the Procrustes distance between the position of that point in the mean shape and in that specimen. Warmer colours denote landmarks having higher displacement from the mean, and cooler colours are more similar to the mean shape. Data and code archived at www.github.com/rnfelice/Dinosaur_Skulls.

To further illustrate the evolutionary mosaicism across the cranial regions and taxa, we calculated the distance from the mean skull shape for each specimen in the 11-module dataset for each landmark (Fig 4). In non-avian theropods, the region of highest phenotypic change is typically the rostrum (Fig 4). In some theropods (*Citipati*, *Tyrannosaurus*, *Allosaurus*, and *Majungasaurus*), the quadrate and quadratojugal are also very different from the average non-avian dinosaur skull shape. In ornithischians, the largest amount of phenotypic change is typically localised to the circumorbital bones and the cranial vault (Fig 4). Notably, ankylosaurs (*Pawpawsaurus* and *Panoplosaurus*) and *Stegosaurus*, like non-avian theropods, exhibit a large amount of phenotypic change in the premaxilla. Unlike theropods, however, the maxilla has low Procrustes distance from the mean in these taxa. Due to its prominent frill, the region with the highest magnitude of change in *Protoceratops* is the posterior parietal. Unexpectedly, the prosauropod *Plateosaurus* most closely resembles the pattern of cranial variation typical of Theropoda, with the greatest deviations from the average dinosaur skull observed in the face and jaw joint. Sauropods, in contrast, exhibit a different pattern. *Camarasaurus* is characterised by a large amount of phenotypic change in the vault, lacrimal, and ventrolateral margin of the skull. In contrast, *Diplodocus*, with its distinctive anteroposteriorly elongate face, has the ventral margin of the premaxilla, maxilla, and jugal as regions of greatest change.

## Discussion

The patterns of cranial shape evolution exhibited by birds do not reflect those of the other dinosaur groups. Despite their ecomorphological diversification, birds display slower rates of evolution than non-avian dinosaurs in almost all cranial regions. The regions of the dinosaur skull that have experienced the highest rates of evolution and achieved the highest disparity differ across clades and largely reflect clade-specific anatomical specialisations, including elaborate skull ornamentations. Whereas the fastest evolving part of the avian skull is the anterior rostrum [29,46], this is not the case for all dinosaurs. Collectively, non-avian dinosaurs have high rates of evolution in the cranial vault and the relative position of the jaw joint.

We propose that these regions have experienced rapid evolutionary change in non-avian dinosaurs, but not birds, for several reasons. First, rapid evolution in the parietal and squamosal are attributed to the diverse cranial ornaments that occur across theropods and marginocephalians. Bony ornamental structures are uncommon in extant birds, with most cranial display in birds being achieved through soft tissue and feathers [41]. However, the few birds with bony crests have previously been shown to exhibit rapid evolution in the skull roof [29]. Ornamental structures, whether they evolve through sexual selection, for species recognition, or for some other function [47,48], are presumably under more intensive phenotypic selection and are expected to show high rates of evolution and high variability. However, the presence of display structures alone cannot explain high rates of vault evolution in non-avian dinosaurs, as many unornamented taxa exhibit rapid phenotypic evolution in this region.

Another possible driver of phenotypic variation and rapid evolutionary change in the vault in non-avian dinosaurs is the structure and function of the adductor chamber. The squamosal and parietal contribute to the borders of the superior temporal fenestra, through which several major jaw muscles pass. These include the *m*. *adductor mandibulae externus profundus*, *medialis*, and *superficialis*, jaw closing muscles that originate on the temporal fossa and temporal bar [49]. The shape of these vault elements thus determines both the size of the jaw adductor apparatus and therefore foraging performance and behaviour. Like the vault, the quadratojugal shows high evolutionary rates and disparity (Fig 3). This element is also linked to food acquisition and processing; as in the squamosal, the dorsoventral length of the quadratojugal relates to the total height of the adductor chamber and thus the length and functional properties of the associated jaw adductor muscles. Furthermore, in some clades (tyrannosaurs, hadrosaurs), the *pterygoideus ventralis* muscle attaches to the jugal, further emphasising the importance of shape variation in this region for jaw function [49].

In non-avian dinosaurs, the quadrate exhibits the highest evolutionary rate in the 11-module dataset (Fig 3) in contrast to birds, which show very low evolutionary rates for the quadrate [29]. The position, orientation, and shape of the articular surface of the quadrate has important implications for mechanical advantage and range of motion of the jaw joint proper and thus jaw function. Similarly, snakes exhibit high disparity and rates in the quadrate, reflecting functional variation in their highly kinetic skulls, especially in comparison to other members of Squamata [42]. The rapid evolution of disparity in the quadrate in non-avian dinosaurs may similarly reflect variation in jaw function. A variety of complex “chewing” mechanics independently evolved across ornithischian clades, including ceratopsians, ankylosaurs, and hadrosaurs, with a wide range of occlusal patterns and power strokes [50–54]. Likewise, feeding mechanics are variable in Theropoda, with hyper-carnivorous, bone-crushing, edentulous, and herbivorous forms [13,37,55,56]. There is much less biomechanical variation in jaw joint function in extant birds, although there is variation in cranial kinesis mechanics within Neoaves [57,58]. As such, the higher rates and disparity of both the adductor chamber region and the jaw joint in non-avian dinosaurs are likely associated with their high variation in jaw biomechanics and food processing strategies.

Variation in food acquisition and processing mechanisms may also have driven differences in rate and disparity in the anterior face across Dinosauria. In birds, the anterior rostrum is the most rapidly evolving and variable part of the skull [29]. However, the mean rate scalar in non-theropod dinosaurs is more than 2 times higher than in avian and non-avian theropods. This theropod/non-theropod split is partly due to high variation in the nasal bone, which is ornamented in some ceratopsians and *Lambeosaurus* or contributes to unusually positioned nares as in sauropods such as *Diplodocus*. However, rapid rostrum evolution in non-theropod dinosaurs is primarily driven by the premaxilla, which evolves slowly in most non-avian theropods but much faster in most ornithischians. The non-avian theropods that do achieve high rates of premaxilla evolution are those with edentulous premaxillae and/or rhamphotheca, including ornithomimosaurs and *Citipati* (S26 Fig and S27 Fig). Similarly, taxa with edentulous premaxillae (hadrosaurs, ceratopsians) have high rates of evolution within Ornithischia.

In birds, the evolution of an edentulous beak is part of a suite of cranial traits, including increased encephalisation and orbit size, that have been linked with the transition to a more paedomorphic cranial phenotype [9,10,21,59–61]. The evolution of this derived neurocranial morphology, emphasising brain size and visual acuity, could have then constrained the potential for variation related to other neurocranial functions, including jaw musculature and bony ornaments. Conversely, non-avian dinosaurs with low encephalisation [62] would not experience this constraint and therefore would be able to rapidly evolve diverse neurocranial and jaw joint phenotypes (although note that some non-avian dinosaurs, like birds, were relatively highly emphasised, see [11]).

These observed differences in rates and disparity among dinosaur groups may be influenced in part by the particular sampling strategy employed in this study. Some clades are under-sampled due to lack of adequately preserved specimens, including Sauropoda and basal members of Avialae. Including additional taxa in future analyses will surely improve our understanding of the tempo and mode of trait evolution. However, our diagnostic analyses of branch length heterogeneity and the relationship between branch length and evolutionary rate suggests that the higher rates observed in non-avian dinosaurs are not an artefact due to gaps in sampling but are in fact biological patterns.

Each cranial region evolves relatively slowly in birds, suggesting that birds, despite some being considered an icon of adaptive radiations, did not achieve their extant cranial diversity through extraordinarily high rates of evolutionary change. The higher rates and disparity of cranial shape evolution in non-avian theropods, especially in the posterior skull (vault, occiput, and pterygoid), starkly contrasts with previous studies comparing disparity and evolutionary rates in the postcranial skeleton of birds and non-avian dinosaurs. Birds have significantly greater disparity in limb proportions [15,19] and higher rates of limb evolution [15,18,22] than non-avian theropods. Similarly, analysis of rates of evolution using discrete morphological characters have demonstrated that the skeleton, as a whole, evolves faster in Avialae than in other theropods [16]. Different conclusions about the relative evolutionary rates of birds in the present analysis and previous studies exemplify the mosaic evolution of birds [29]. The modular organisation of the avian skeleton allows different regions of the body to evolve at different rates in response to different selective pressures, and different tempo of evolution between regions of the theropod skeleton have been demonstrated previously [19,20,63]. While some previous analyses of phenotypic evolution in theropods have included cranial traits and concluded that birds evolve significantly faster than other theropods, these works did not partition traits according to region (e.g., cranial versus forelimb versus hindlimb) [16,18]. As such, high rates of evolution in the limbs may overwhelm low rates of evolution in the skull, generating an overall pattern of rapid phenotypic change in birds.

As birds were experiencing high rates of evolution in the postcranial skeleton and using their newfound variation in limb morphology to exploit a variety of habitats, their cranial evolution had slowed down relative to other dinosaurs. Whether this is related to the biomechanical demands of aerial locomotion or encephalisation, developmental factors caused by reorganisation of the palate and vault, or phylogenetic inertia, birds failed to achieve the high rates of cranial evolution observed in non-avian dinosaurs. While the wings [22,63], hindlimb [64], and axial skeleton [65–67] rapidly achieved high disparity, cranial diversity decelerated compared to the diversity that was once present across Dinosauria, reinforcing that avian morphology and disparity are the product of mosaic evolution.

## Methods

### Taxonomic sampling

Sampled taxa include 354 extant birds, 1 extinct bird, and 36 extinct non-avian dinosaurs. Extant taxa were selected to represent the breadth of cranial diversity and over 80% of extant families. Fossil specimens were selected on the quality of their preservation, with a mostly complete, articulated, and 3D skull. Repositories for each specimen, along with specimen ID numbers, are listed in S4 Table.

### Geometric morphometric data

Digital 3D models of specimens were generated via CT scanning and surface scanning. We digitised anatomical landmarks and semi-landmark curves on the surface of each specimen using IDAV Landmark [68], defining the boundaries of bones or clusters of adjacent bones (S5 Table). Following established procedures [29,32,33], we digitised these same landmarks and semi-landmarks on a generic hemisphere template, in addition to surface semi-landmarks within each of the regions bordered by semi-landmark curves. In non-avian dinosaurs, we identified the boundaries of 16 regions: the dorsolateral surface of the maxilla, the dorsolateral surface of the premaxilla, the ventral surface of the maxilla, the ventral surface of the premaxilla, nasal, frontal, parietal, squamosal, palatine, pterygoid, sphenoid, the articular surface of the quadrate, the occiput, postorbital, lacrimal + prefrontal, and the jugal + quadratojugal. When present, the rostral bone was included as part of the “premaxilla” module. Surface semi-landmarks were then projected on to the surface of each specimen based on the correspondences between the landmarks and semi-landmark curves on the template and target specimen, generating a high-dimensional landmark configuration for each specimen (S1 Fig).

Because of the fusion of cranial elements in birds, not all anatomical landmarks and semi-landmark curves can be digitised in all taxa. For example, the parietal, frontal, and squamosal are distinct elements in non-avian dinosaurs, but their borders are typically indistinguishable in adult birds. To enable comparison between birds and non-avian dinosaurs, we projected the same surface semi-landmarks onto birds and non-avian dinosaurs using two separate templates. The initial template with 16 anatomical regions was generated based on the bones and sutures present in non-avian dinosaurs (S4 Table). We then modified this template by deleting the anatomical landmarks and curves and retaining the surface semi-landmarks, then digitising new anatomical landmarks and curves around clusters of points [33]. Birds and dinosaurs were patched separately; then the curves and landmarks were removed from each, and the surfaces semi-landmarks were combined to form a complete dataset with corresponding landmarks.

Even though we selected only the most complete fossil skulls, only 9 specimens preserved every element of interest. We analysed 3 subsets in order to maximise taxonomic and anatomical breath:

1. The non-avian dinosaur specimens that adequately preserve lateral and dorsal sides of the skull (*n* = 27); this dataset includes maxilla, premaxilla, nasal, frontal, parietal, squamosal, jugal + quadratojugal, postorbital, and lacrimal + prefrontal and is referred to hereafter as the “9-module dataset”
2. The non-avian dinosaur specimens that preserve all the regions in the 9-module dataset, as well as the occipital region and the articular surface of the quadrate (*n* = 19), referred to hereafter as the 11-module dataset
3. Birds and non-avian dinosaurs combined, with each cranial module analysed individually. This allows for the greatest number of fossil specimens to be included for comparison with modern birds. The non-avian dinosaur specimens not included in the 9-module dataset were used in per-module analyses of non-avian dinosaurs only.

Our hypothesis of the modular organisation of the skull is based on the empirically supported hypothesis for extant birds and consists of 6 modules: the rostrum (dorsal maxilla, premaxilla, nasal, jugal region), palate (ventral maxilla, premaxilla, palatine), cranial vault (frontal, parietal, squamosal), occipital region, basisphenoid, and quadrate + pterygoid [29]. We also analysed the quadrate and pterygoid separately. Inclusion of non-avian dinosaurs in each dataset is indicated in S4 Table.

### Phylogenetic hypotheses

The major phylogenetic relationships among dinosaurs are currently debated. The traditional view on dinosaur relationships has split the group into 2 major groups, Saurischia (Theropoda and Sauropoda) and Ornithischia. However, some recent analyses have supported an alternative hypothesis, with Theropoda and Ornithischia forming a monophyletic clade (Ornithoscelida) that is sister to Saurischia + Herrerasauridae [69,70]. As such, we carried out all phylogenetic comparative methods using 2 different topologies, one with the traditional Saurischia + Ornithischia hypothesis and one with the Ornithoscelida hypothesis. To generate these topologies, we utilised the procedure in [14] to time-calibrate a topology corresponding to the “traditional” hypothesis to stratigraphy with the minimum branch lengths (MBL) method. We then grafted the time-calibrated extant bird phylogeny from reference [3] onto this tree. We then created an “Ornithoscelida hypothesis” tree by manually manipulating the branching of the basal nodes of the traditional hypothesis tree using Mesquite version 3.6 [71]. Finally, we evaluated the effects of tip-dating methods on rate reconstructions by applying the fossilised birth-death (FBD) model [72], implemented in MrBayes 3.2.7a [73], as an alternative approach to calibrate the traditional topology. Using the “createMrBayesTipDatingNexus” function in the R package “paleotree” [74], we generated a Nexus file for input into MrBayes. Beginning with the undated fossil tree from reference [14], we constrained the topology so that only branch lengths, rather than topologies, were estimated by MrBayes. We applied a uniform prior for the root age from 247.1 to 257.1 million years, as well as uniform priors for taxon dates using the same stratigraphic data used for the MBL method. Default settings were used for all other model priors. The analysis was conducted for 2 runs of 4 chains each, with 100,000,000 generations and a burn-in of 50%. We summarised the results, generating a single maximum clade credibility tree, using the “obtainDatedPosteriorTreesMrB” function in “paleotree” [74]. Finally, we grafted the extant bird topology on to the FBD to create the final, dated phylogeny. BayesTraits variable rates analyses with birds and non-avian dinosaurs were carried out with all topologies. However, macroevolutionary patterns are nearly identical under all approaches, therefore only the results of the traditional topology—dated using MBLs—are included in the main text. Full results for all phylogenetic hypotheses are presented in the S2–S22 Figs.

### Data analysis

We generated morphospace plots (S47 Fig) to investigate the patterns of cranial variation across birds and dinosaurs [75]. We conducted a single Procrustes alignment for all specimens preserving the rostrum, vault, and occiput (22 non-avian dinosaurs and all birds). Birds and non-avian dinosaurs occupy distinct but overlapping regions of morphospace in the first 4 principal component axes, which together account for 66% of the cumulative variance. Surprisingly, on PC axes 1 and 2, *Diplodocus* occupies a region or morphospace closer to birds, unlike theropod taxa that are more closely related to birds. This is a result of the elongate face and more posteriorly positioned nares in *Diplodocus* superficially resembling birds. Similarly, the highly domed cranial vault of pachycephalosaurs generates similarities to the highly encephalised birds.

We quantified rates of skull shape evolution across Dinosauria using BayesTraitsV3 (http://www.evolution.rdg.ac.uk/). Preliminary BayesTraits analyses confirmed that variable-rates models [38] were favoured over single-rate modules for all modules and phylogenetic hypotheses (Bayes factor > 100). As such, we present only the results of variable-rates models here. We conducted separate Procrustes fits for each module and conducted phylogenetic principal components analysis for each [76]. Because different fossil specimens preserve different subset of the regions comprising the skull, this separate Procrustes fit approach allows us to maximise the taxonomic sample for each region. We used the PC scores from the PC axes that account for 95% of the cumulative variance for each module to reduce the dimensionality of the data while still quantifying shape as a multidimensional trait. We carried out BayesTraits analyses for birds and non-avian dinosaurs together for each of the 7 modules present in birds (rostrum, palate, cranial vault, occipital region, basisphenoid, quadrate, and pterygoid). In addition, we fit variable rates models for each of the modules present in non-avian dinosaurs. For each module and for all phylogenetic hypotheses, we fit variable-rates BM with 100,000,000 iterations and a burn-in of 12,500,000. Traits were considered correlated for the purposes of model construction in BayesTraits. Each analysis was carried out 3 times to confirm that the Markov Chain reached convergence, which was confirmed using Gelman and Rubin’s convergence diagnostic test statistic, implemented as the “gelman.diag” function in the R package “coda” [77]. The run with the highest mean marginal likelihood was retained for interpretation.

To compare evolutionary rates between birds and other dinosaur groups, we calculated the mean rate scalar per branch for each group and each module (Fig 2). For each tree in the post-burn-in posterior distribution of each analysis, we extracted the per-branch rate scalars using the R package BTRTools (http://github.com/hferg/BTRTools). We then extracted the subtrees for birds, non-avian theropods, and non-theropod dinosaurs and calculated the mean rate scalar for that subtree, then divided this value by total subtree height to produce time-corrected mean rate scalar (Fig 2). To test whether these groups exhibit significantly different rates, we compared the distribution of mean rate scalars for each group using nonparametric *t* tests (Wilcoxon tests).

Because the number of bird species is much greater than the number of non-avian dinosaurs, we performed a subsampling analysis to confirm that the rate of evolution in each group was robust to sample size and to the effects of individual branches with extremely high or low rates. We randomly selected one species per avian order and removed all other birds from the dataset, resulting in 40 extant birds and up to 37 extinct dinosaurs. Using this subsampled dataset, we generated a new phylogenetic principal components analysis and again modelled variable-rate evolution using BayesTraits with the same parameters as with the full dataset. This procedure was repeated for 100 iterations for each skull region. To summarise these results, we extracted the mean branch rate scalars from the posterior distribution from each iteration (S37 Fig). Because overall patterns were congruent among phylogenetic hypotheses, we carried out these subsampling analyses only using the MBL-dated traditional Dinosauria phylogenetic hypothesis.

As noted earlier, separate Procrustes alignments for each module were applied so that every specimen that preserved each structure could be used, regardless of the preservation of the rest of the skull. Therefore, only the shape of each structure was considered and not its relative position in the skull. However, for some structures (e.g., the quadrate), position was hypothesised to be a significant aspect of variation. To quantify this, we also estimated variable-rates models on the rostrum, vault, occipital region, and quadrate with a global Procrustes alignment for all of these regions (S38 Fig), thus calculating the same metrics while taking relative position into account.

Whereas these evolutionary models were estimated using phylogenetic principal components scores of shape, we also sought to analyses rates of evolution from the landmark configurations directly. This is possible using the σ_mult_ metric implemented in the “geomorph” R package [39,78]. This method quantifies the amount of shape change that has occurred along the length of the phylogeny with a single value. In addition, this method can be used to compare the relative rates of evolution among subsets or modules from a single landmark configuration. However, this metric assumes an equal-rates BM model, whereas BayesTraits modelling confirms that these traits evolve with variable rates. To confirm whether results of σ_mult_ tests with these data are robust to this violation of the assumptions of the method, we calculated per-group rates for each region using equal rates with σ_mult_ (S39 Fig) and compared these results to the analogous BayesTraits test (Fig 2). Using the “compare.evol.rates” function in the “geomorph” R package, we quantified the evolutionary rate of each group for each region, using the MBL-dated traditional hypothesis and 999 simulations to assess significance (S1 Table). As single-rate approaches may be particularly susceptible to outlier taxa (i.e., individual branches with unusually fast or slow rates), we repeated this analysis using the same subsampling procedure described earlier (Fig 2) for both the MBL-dated traditional topology phylogenetic hypothesis (S39 Fig). We also carried out the σ_mult_ analyses with the FBD-dated phylogenetic tree (S40 Fig) and global superimposition (S42 Fig). Again, the patterns are similar to those observed with the MBL-dated tree and with local superimpositions. Birds do not have the highest σ_mult_ in any region of the skull. While absolute magnitudes of these rates vary between analyses, the relative rates are the same (e.g., in the rostrum, non-theropod dinosaurs are always fastest, birds second fastest, and non-avian theropods slowest). The relative branch lengths in these topologies reveals that the MBL-dated tree reconstructs shorter branch lengths at the root of Coelurosauria, Maniraptora, and Ornithomimosauria compared to the FBD-dated tree, resulting in faster rates of evolution in non-avian theropods using the FBD-dated tree than in the MBL-dated tree. Taken together, the consistency between the results of clade-wise comparisons of rates with σ_mult_ and BayesTraits variable-rates models demonstrates that these findings are robust to model choice and method.

We further visualised fine-scale regional variation in evolutionary rates by quantifying σ_mult_ for each landmark in the 9-module and 11-module datasets using the functions “per_lm_rates” and “per_lm_variance” in the R package “hot.dots” [79]. This is equivalent to using the “compare.multi.evol.rates” function in “geomorph” and assigning every landmark to its own module [39]. As with evolutionary rate, we measured disparity (Procrustes variance) for each module and for each landmark in the 9-module and 11-module datasets. We illustrated regional variation in evolutionary rate and variance by plotting the landmarks as spheres on an example specimen with the colour of each landmark associated with the log-transformed rate (Fig 3A and 3C) or log-transformed variance (Fig 3B and 3D). We illustrated which regions of the skull differ among taxa by calculating per-landmark Procrustes distance between each specimen and the mean landmark configuration using the 11-module dataset, and then plotting this mean shape with landmark colours proportional to these distances (Fig 4), using functions from the R package “landvR” [80]. For each region present in birds, we compared Procrustes variance among birds, non-avian theropods, and non-theropod dinosaurs using the “morphol.disparity” function in “geomorph” to test for differences in disparity among groups.

### Evaluating the influence of sampling and tip-dating approaches

To validate our results, we performed 2 post hoc tests to examine whether rate estimations is strongly influenced by artefacts related to sampling and tip-dating strategies. First, we tested whether the bird and non-avian dinosaurs show different distributions of branch lengths in the time-calibrated phylogeny, as disproportionately long branches could artificially decrease mean rates. With our interspecific sampling, birds indeed have shorter branch lengths than non-avian dinosaurs (S45 Fig; two-sample *t* test: *t* = −4.3918, df = 73.732, *p* = 3.6 × 10^−5^). However, when the avian phylogeny is subsampled to include the same number of taxa as our sampling of non-avian dinosaurs, the branch lengths are not significantly different between these two groups (*t* = −0.54268, df = 72.032, *p* = 0.589). Because the results of σ_mult_ analyses with and without subsampling are largely congruent, these findings imply that branch length distributions are not skewing rate calculations. To test whether the evolutionary rates estimated using BayesTraits were biased by the estimated branch lengths, we regressed log-transformed mean rate from variable-rates BM model onto the lengths of each branch in the 3 phylogenetic hypotheses (S46 Fig). The slopes of these regressions are similar for all 3 trees, although overall rates are lower in the FBD-dated tree, causing a lower intercept. There is not a significant correlation between these parameters (*R*^2^ = 0.003, *p* = 0.07), further supporting the conclusion that rate heterogeneity is not primarily driven by differences in branch lengths.

## Supporting information

S1 Fig High-dimension Geometric Morphometric Approach. Anatomical landmarks and semi-landmark curves were digitised onto 3D surface meshes, and surface semi-landmarks were projected onto the skull using the Morpho R package (A, lateral; B, dorsal; C, ventral). Landmark configurations for dinosaurs were partitioned into a total of 16 anatomical regions (D, lateral; E, dorsal; F, ventral). Non-avian dinosaurs were compared to non-avian dinosaurs by re-partitioning landmarks into regions that can be distinguished in both birds and non-avian dinosaurs (G, lateral; H, dorsal; I, ventral). Landmark configuration illustrated on *Erlikosaurus andrewsi* (IGM 100/111). Data and code archived at www.github.com/rnfelice/Dinosaur_Skulls.
(PDF)

S2 Fig Estimation of rates of rostrum evolution (traditional phylogenetic hypothesis). Modelled using a variable-rates BM model of evolution. Data and code archived at www.github.com/rnfelice/Dinosaur_Skulls.
(PDF)

S3 Fig Estimation of rates of rostrum evolution (Ornithoscelida hypothesis). Modelled using a variable-rates BM model of evolution. Data and code archived at www.github.com/rnfelice/Dinosaur_Skulls.
(PDF)

S4 Fig Estimation of rates of rostrum evolution (FBD-dated tree). Modelled using a variable-rates BM model of evolution. Data and code archived at www.github.com/rnfelice/Dinosaur_Skulls.
(PDF)

S5 Fig Estimation of rates of cranial vault evolution (traditional phylogenetic hypothesis). Modelled using a variable-rates BM model of evolution. Data and code archived at www.github.com/rnfelice/Dinosaur_Skulls.
(PDF)

S6 Fig Estimation of rates of cranial vault evolution (Ornithoscelida hypothesis). Modelled using a variable-rates BM model of evolution. Data and code archived at www.github.com/rnfelice/Dinosaur_Skulls.
(PDF)

S7 Fig Estimation of rates of cranial vault evolution (FBD-dated tree). Modelled using a variable-rates BM model of evolution. Data and code archived at www.github.com/rnfelice/Dinosaur_Skulls.
(PDF)

S8 Fig Estimation of rates of occiput evolution (traditional phylogenetic hypothesis). Modelled using a variable-rates BM model of evolution. Data and code archived at www.github.com/rnfelice/Dinosaur_Skulls.
(PDF)

S9 Fig Estimation of rates of occiput evolution (Ornithoscelida hypothesis). Modelled using a variable-rates BM model of evolution. Data and code archived at www.github.com/rnfelice/Dinosaur_Skulls.
(PDF)

S10 Fig Estimation of rates of occiput evolution (FBD-dated tree). Modelled using a variable-rates BM model of evolution. Data and code archived at www.github.com/rnfelice/Dinosaur_Skulls.
(PDF)

S11 Fig Estimation of rates of palate evolution (traditional phylogenetic hypothesis). Modelled using a variable-rates BM model of evolution. Data and code archived at www.github.com/rnfelice/Dinosaur_Skulls.
(PDF)

S12 Fig Estimation of rates of palate evolution (Ornithoscelida hypothesis). Modelled using a variable-rates BM model of evolution. Data and code archived at www.github.com/rnfelice/Dinosaur_Skulls.
(PDF)

S13 Fig Estimation of rates of palate evolution (FBD-dated tree). Modelled using a variable-rates BM model of evolution. Data and code archived at www.github.com/rnfelice/Dinosaur_Skulls.
(PDF)

S14 Fig Estimation of rates of quadrate evolution (traditional phylogenetic hypothesis). Modelled using a variable-rates BM model of evolution. Data and code archived at www.github.com/rnfelice/Dinosaur_Skulls.
(PDF)

S15 Fig Estimation of rates of quadrate evolution (Ornithoscelida hypothesis). Modelled using a variable-rates BM model of evolution. Data and code archived at www.github.com/rnfelice/Dinosaur_Skulls.
(PDF)

S16 Fig Estimation of rates of quadrate evolution (FBD-dated tree). Modelled using a variable-rates BM model of evolution. Data and code archived at www.github.com/rnfelice/Dinosaur_Skulls.
(PDF)

S17 Fig Estimation of rates of pterygoid evolution (traditional phylogenetic hypothesis). Modelled using a variable-rates BM model of evolution. Data and code archived at www.github.com/rnfelice/Dinosaur_Skulls.
(PDF)

S18 Fig Estimation of rates of pterygoid evolution (Ornithoscelida hypothesis). Modelled using a variable-rates BM model of evolution. Data and code archived at www.github.com/rnfelice/Dinosaur_Skulls.
(PDF)

S19 Fig Estimation of rates of pterygoid evolution (FBD-dated tree). Modelled using a variable-rates BM model of evolution. Data and code archived at www.github.com/rnfelice/Dinosaur_Skulls.
(PDF)

S20 Fig Estimation of rates of ventral sphenoid region evolution (traditional phylogenetic hypothesis). Modelled using a variable-rates BM model of evolution. Data and code archived at www.github.com/rnfelice/Dinosaur_Skulls.
(PDF)

S21 Fig Estimation of rates of ventral sphenoid region evolution (Ornithoscelida hypothesis). Modelled using a variable-rates BM model of evolution. Data and code archived at www.github.com/rnfelice/Dinosaur_Skulls.
(PDF)

S22 Fig Estimation of rates of ventral sphenoid region evolution (FBD-dated tree). Modelled using a variable-rates BM model of evolution. Data and code archived at www.github.com/rnfelice/Dinosaur_Skulls.
(PDF)

S23 Fig Estimation of rates of frontal bone evolution. Modelled using the (A) traditional phylogenetic hypothesis and (B) Ornithoscelida hypothesis under a variable-rates BM model of evolution. Data and code archived at www.github.com/rnfelice/Dinosaur_Skulls.
(PDF)

S24 Fig Estimation of rates of parietal bone evolution. Modelled using the (A) traditional phylogenetic hypothesis and (B) Ornithoscelida hypothesis under a variable-rates BM model of evolution. Data and code archived at www.github.com/rnfelice/Dinosaur_Skulls.
(PDF)

S25 Fig Estimation of rates of squamosal bone evolution. Modelled using the (A) traditional phylogenetic hypothesis and (B) Ornithoscelida hypothesis under a variable-rates BM model of evolution. Data and code archived at www.github.com/rnfelice/Dinosaur_Skulls.
(PDF)

S26 Fig Estimation of rates of dorsal premaxilla bone evolution. Modelled using the (A) traditional phylogenetic hypothesis and (B) Ornithoscelida hypothesis under a variable-rates BM model of evolution. Data and code archived at www.github.com/rnfelice/Dinosaur_Skulls.
(PDF)

S27 Fig Estimation of rates of ventral premaxilla bone evolution. Modelled using the (A) traditional phylogenetic hypothesis and (B) Ornithoscelida hypothesis under a variable-rates BM model of evolution. Data and code archived at www.github.com/rnfelice/Dinosaur_Skulls.
(PDF)

S28 Fig Estimation of rates of dorsal maxilla bone evolution. Modelled using the (A) traditional phylogenetic hypothesis and (B) Ornithoscelida hypothesis under a variable-rates BM model of evolution. Data and code archived at www.github.com/rnfelice/Dinosaur_Skulls.
(PDF)

S29 Fig Estimation of rates of ventral maxilla bone evolution. Modelled using the (A) traditional phylogenetic hypothesis and (B) Ornithoscelida hypothesis under a variable-rates BM model of evolution. Data and code archived at www.github.com/rnfelice/Dinosaur_Skulls.
(PDF)

S30 Fig Estimation of rates of dorsal nasal bone evolution. Modelled using the (A) traditional phylogenetic hypothesis and (B) Ornithoscelida hypothesis under a variable-rates BM model of evolution. Data and code archived at www.github.com/rnfelice/Dinosaur_Skulls.
(PDF)

S31 Fig Estimation of rates of jugal and quadratojugal bone evolution. Modelled using the (A) traditional phylogenetic hypothesis and (B) Ornithoscelida hypothesis under a variable-rates BM model of evolution. Data and code archived at www.github.com/rnfelice/Dinosaur_Skulls.
(PDF)

S32 Fig Estimation of rates of occiput evolution. Modelled using the (A) traditional phylogenetic hypothesis and (B) Ornithoscelida hypothesis under a variable-rates BM model of evolution. Data and code archived at www.github.com/rnfelice/Dinosaur_Skulls.
(PDF)

S33 Fig Estimation of rates of ventral sphenoid region evolution. Modelled using the (A) traditional phylogenetic hypothesis and (B) Ornithoscelida hypothesis under a variable-rates BM model of evolution. Data and code archived at www.github.com/rnfelice/Dinosaur_Skulls.
(PDF)

S34 Fig Estimation of rates of quadrate bone evolution. Modelled using the (A) traditional phylogenetic hypothesis and (B) Ornithoscelida hypothesis under a variable-rates BM model of evolution. Data and code archived at www.github.com/rnfelice/Dinosaur_Skulls.
(PDF)

S35 Fig Estimation of rates of pterygoid bone evolution. Modelled using the (A) traditional phylogenetic hypothesis and (B) Ornithoscelida hypothesis under a variable-rates BM model of evolution. Data and code archived at www.github.com/rnfelice/Dinosaur_Skulls.
(PDF)

S36 Fig Estimation of rates of palatine bone evolution. Modelled using the (A) traditional phylogenetic hypothesis and (B) Ornithoscelida hypothesis under a variable-rates BM model of evolution. Data and code archived at www.github.com/rnfelice/Dinosaur_Skulls.
(PDF)

S37 Fig Comparison of per-group evolutionary rate scalars with subsampled data. Birds were randomly subsampled to 1 species per extant order for 100 iterations. Subsampled data were used to estimate variable-rates BM models, and then mean rate was calculated for each group and for each iteration and scaled by dividing by the sum of the branch lengths in the corresponding time-scaled subtree. Models used the traditional Dinosauria phylogenetic topology (Saurischia and Ornithischia as sister clades). Mean rate scalars were compared between groups modelled using nonparametric *t* tests; significantly different distributions are indicated with ****p < 0.00005. Data and code archived at www.github.com/rnfelice/Dinosaur_Skulls.
(PDF)

S38 Fig Comparison of per-group evolutionary rate scalars with global Procrustes superimposition. Mean rate scalar was calculated from the mean of the rate scalars for the indicated group in the post-burn-in posterior distribution of the variable-rates evolutionary model estimated modelled using BayesTraits. Mean rates were scaled by dividing by the sum of the branch lengths in the corresponding time-scaled subtree. Models used the traditional Dinosauria phylogenetic topology (Saurischia and Ornithischia as sister clades). Mean rate scalars were compared between groups Modelled using nonparametric *t* tests; significantly different distributions are indicated with ****p < 0.00005. Data and code archived at www.github.com/rnfelice/Dinosaur_Skulls.
(PDF)

S39 Fig Comparison of per-group evolutionary rates calculated using a phylogeny dated with the MBL method. Birds do not have the highest rates of evolution in any cranial region. Rate of evolution was calculated using the σ_mult_ metric [39]. Because overall sampling for birds was higher than for non-avian dinosaurs, we subsampled the birds to 1 species per order for 100 iterations. Rates were compared between groups using nonparametric *t* tests; significantly different distributions are indicated with ****p < 0.00005. Data and code archived at www.github.com/rnfelice/Dinosaur_Skulls.
(PDF)

S40 Fig Comparison of per-group evolutionary rates calculated using a phylogeny dated with the FBD model. Birds do not have the highest rates of evolution in any cranial region. Rate of evolution was calculated using the σ_mult_ metric [39]. Because overall sampling for birds was higher than for non-avian dinosaurs, we subsampled the birds to 1 species per order for 100 iterations. Rates were compared between groups using nonparametric *t* tests; significantly different distributions are indicated with ****p < 0.00005). Data and code archived at www.github.com/rnfelice/Dinosaur_Skulls.
(PDF)

S41 Fig Evaluating the effects of influential taxa on per-group evolutionary rates. Rate of evolution was calculated using the σ_mult_ metric [39]. In addition to birds being subsampled to one species per order as in S38 Fig, one species was randomly removed from each dinosaur group in each of the 100 iterations. Rates were compared between groups using nonparametric *t* tests; significantly different distributions are indicated with ****p < 0.00005). Data and code archived at www.github.com/rnfelice/Dinosaur_Skulls.
(PDF)

S42 Fig Comparison of per-group evolutionary rates with global Procrustes superimposition. Rates calculated using the FBD-dated phylogenetic hypothesis. The high rates of evolution in the non-avian dinosaur quadrate observed in Fig 2 are likely a result of changes in the relative position of the jaw joint relative to the rest of the skull. The results presented in S37 Fig utilise separate Procrustes superimpositions for each region, and thus do not preserve differences in position among skull region. Here, under global Procrustes imposition, non-avian dinosaurs have significantly higher rates of quadrate evolution compared to birds. Rates were compared between groups using nonparametric *t* tests; significantly different distributions are indicated with ****p < 0.00005. Data and code archived at www.github.com/rnfelice/Dinosaur_Skulls.
(PDF)

S43 Fig Rate, disparity, and integration. Bivariate plots of disparity (Procrustes variance), mean evolutionary rate, and within-partition trait correlation (integration). Values were calculated for the 9-module dataset (A–C) and the 11-module dataset (D–F). Disparity is corrected by the number of landmarks and sliding semi-landmarks in the region. Within-module correlation values derived from ref [33]. Rates calculated using the traditional dinosaur phylogenetic topology, dated with the MBL method. Data and code archived at www.github.com/rnfelice/Dinosaur_Skulls.
(PDF)

S44 Fig Phenotypic disparity across groups. Calculated using the morphol.disparity function in the geomorph R package. Each cranial region except the quadrate is less disparate in birds than in non-avian groups. Data and code archived at www.github.com/rnfelice/Dinosaur_Skulls.
(PDF)

S45 Fig Comparison of branch lengths among groups. The extant bird portion of the phylogeny has shorter branch lengths due to denser sampling, but this effect is removed by the subsampling procedure. Data and code archived at www.github.com/rnfelice/Dinosaur_Skulls.
(PDF)

S46 Fig The relationship between branch length and log-transformed rate. Linear regression reveals that the relationship between rate and branch length is not significant (*R*^2^ = 0.003, *p* = 0.07). Data and code archived at www.github.com/rnfelice/Dinosaur_Skulls.
(PDF)

S47 Fig Cranial morphospace for the rostrum, vault, and occipital regions. The first 4 principal components axes explain a cumulative 66% of the total variance. Grey silhouettes represent the reconstructed shape at the corresponding region of morphospace. Birds and non-avian dinosaurs largely occupy distinct regions of morphospace. *Diplodocus*, with its elongate rostrum and posteriorly positioned nares, is convergent with birds with high PC axis 1 and moderate PC axis 2 scores. The domed skulls of pachycephalosaurs cause them to group with birds with high negative PC 1 scores Data and code archived at www.github.com/rnfelice/Dinosaur_Skulls. PC, principal component
(PDF)

S1 Table Evolutionary rates for each cranial region compared across groups. Calculated with the full dataset using the traditional dinosaur phylogenetic topology dated with the MBL method. Shaded cells indicate significantly faster rate than birds.
(PDF)

S2 Table Phenotypic disparity of each cranial region. Calculated using the morphol.disparity function in the geomorph R package.
(PDF)

S3 Table Significance values for pairwise comparisons of disparity. Calculated using the morphol.disparity function in the geomorph R package.
(PDF)

S4 Table Taxonomic sampling. Specimen number and museum collection data for all sampled taxa.
(XLSX)

S5 Table Landmarking procedure. Anatomical descriptions of landmark locations.
(DOCX)

## Abbreviations

2D: two-dimensional
3D: three-dimensional
BM: Brownian Motion
FBD: fossilised birth-death
MBL: minimum branch lengths
