# Supplementary material for: Decelerated dinosaur skull evolution with the origin of birds
Source: PLoS Biol. 2020 Aug 18;18(8):e3000801. doi: 10.1371/journal.pbio.3000801 (PMC7437466; doi:10.1371/journal.pbio.3000801)
Supplement: S1 Fig — Anatomical landmarks and semi-landmark curves were digitised onto 3D surface meshes, and surface semi-landmarks were projected onto the skull using the Morpho R package (A, lateral; B, dorsal; C, ventral). Landmark configurations for dinosaurs were partitioned into a total of 16 anatomical regions (D, lateral; E, dorsal; F, ventral). Non-avian dinosaurs were compared to non-avian dinosaurs by re-partitioning landmarks into regions that can be distinguished in both birds and non-avian dinosaurs (G, lateral; H, dorsal; I, ventral). Landmark configuration illustrated on Erlikosaurus andrewsi (IGM 100/111). Data and code archived at www.github.com/rnfelice/Dinosaur_Skulls. (PDF) [file pbio.3000801.s001.pdf]

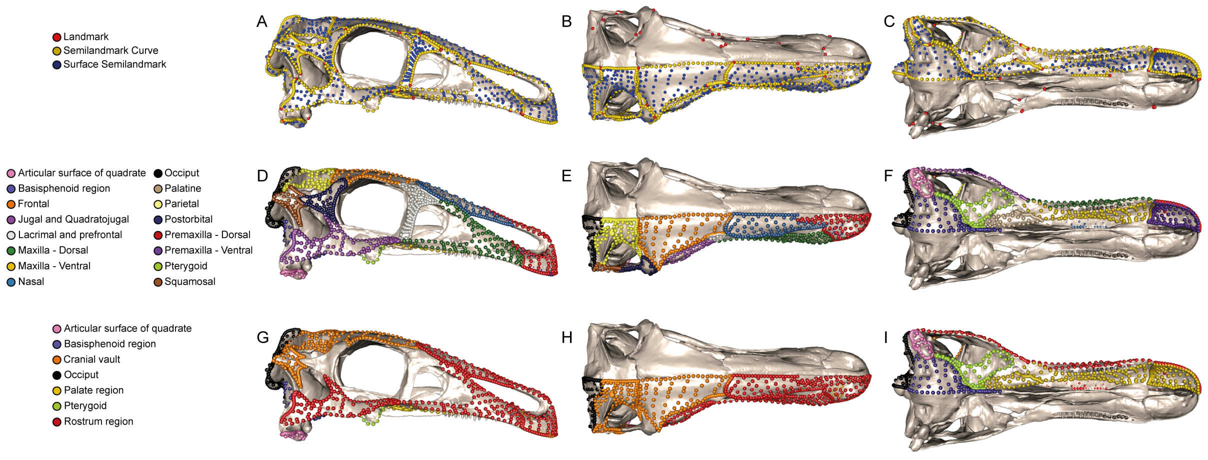

S1 Fig. High-dimension Geometric Morphometric Approach. Anatomical landmarks and semilandmark curves were digitized onto 3D surface meshes and surface semilandmarks were projected on to the skull using the Morpho R package (A, lateral; B, dorsal; C, ventral). Landmark configurations for dinosaurs were partitioned into a total of 16 atomical regions (D, lateral; E, dorsal; F, ventral). Non-avian dinosaurs were compared to non-avian dinosaurs by re-partioning landmarks into regions that can be distinguished in both birds and non-avian dinosaurs (G, lateral; H, dorsal; I, ventral). Landmark configuration illustrated on *Erlikosaurus andrewsi* (IGM 100/111). Data and code archived at www.github.com/rnfelice/Dinosaur_Skulls.
