# Supplementary material for: Decelerated dinosaur skull evolution with the origin of birds
Source: PLoS Biol. 2020 Aug 18;18(8):e3000801. doi: 10.1371/journal.pbio.3000801 (PMC7437466; doi:10.1371/journal.pbio.3000801)
Supplement: S25 Fig — Modelled using the (A) traditional phylogenetic hypothesis and (B) Ornithoscelida hypothesis under a variable-rates BM model of evolution. Data and code archived at www.github.com/rnfelice/Dinosaur_Skulls. (PDF) [file pbio.3000801.s025.pdf]

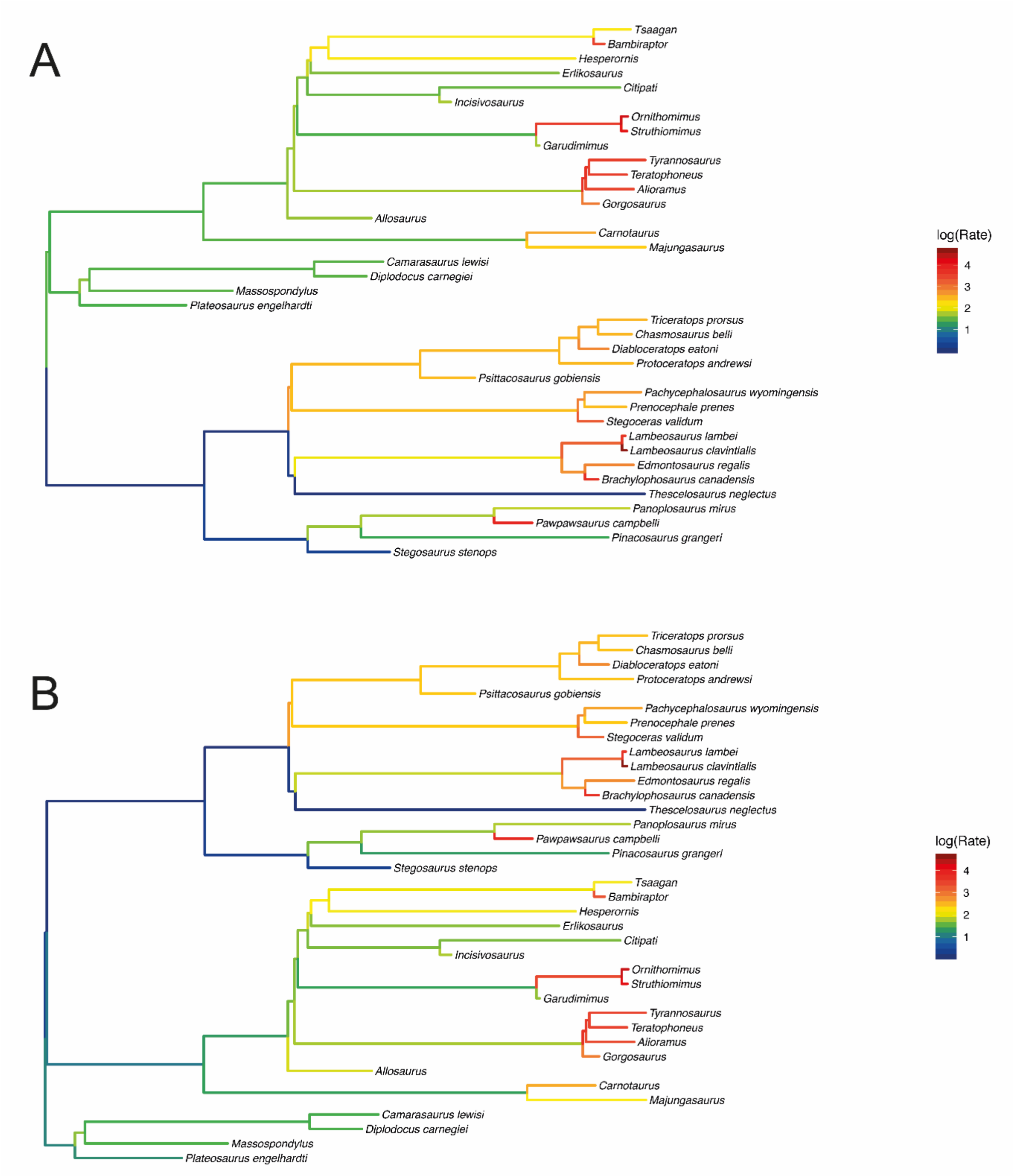

S25 Fig. Estimation of rates of squamosal bone evolution. Modelled using the (A) traditional phylogenetic hypothesis and (B) Ornithoscelida hypothesis under a variable-rates Brownian motion model of evolution. Data and code archived at www.github.com/rnfelice/Dinosaur_Skulls.
