# Supplementary material for: Decelerated dinosaur skull evolution with the origin of birds
Source: PLoS Biol. 2020 Aug 18;18(8):e3000801. doi: 10.1371/journal.pbio.3000801 (PMC7437466; doi:10.1371/journal.pbio.3000801)
Supplement: S37 Fig — Birds were randomly subsampled to 1 species per extant order for 100 iterations. Subsampled data were used to estimate variable-rates BM models, and then mean rate was calculated for each group and for each iteration and scaled by dividing by the sum of the branch lengths in the corresponding time-scaled subtree. Models used the traditional Dinosauria phylogenetic topology (Saurischia and Ornithischia as sister clades). Mean rate scalars were compared between groups modelled using nonparametric t tests; significantly different distributions are indicated with ****p < 0.00005. Data and code archived at www.github.com/rnfelice/Dinosaur_Skulls. (PDF) [file pbio.3000801.s037.pdf]

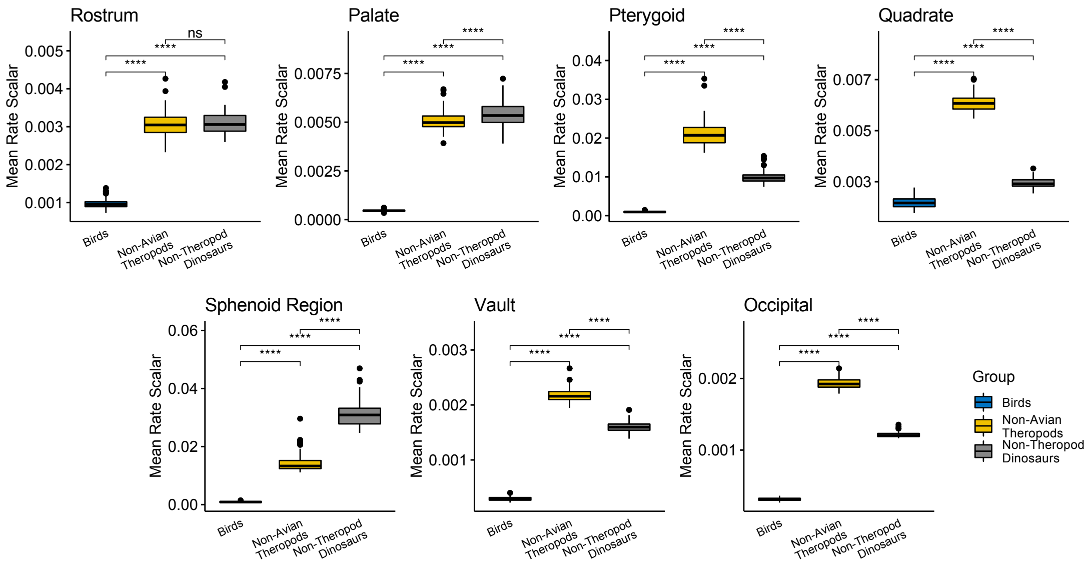

S37 Fig. Comparison of per-group evolutionary rate scalars with subsampled data. Birds were randomly subsampled to 1 species per extant order for 100 iterations. Subsampled data was used to estimate variable-rates Brownian motion models, then mean rate was calculated for each group and for each iteration and scaled by dividing by the sum of the branch lengths in the corresponding time-scaled subtree. Models used the traditional Dinosauria phylogenetic topology (Saurischia and Ornithischia as sister clades). Mean rate scalars were compared between groups Modelled using non-parametric t-tests; significantly different distributions are indicated with **** (p<0.00005). Data and code archived at www.github.com/rnfelice/Dinosaur_Skulls.
