# Supplementary material for: Decelerated dinosaur skull evolution with the origin of birds
Source: PLoS Biol. 2020 Aug 18;18(8):e3000801. doi: 10.1371/journal.pbio.3000801 (PMC7437466; doi:10.1371/journal.pbio.3000801)
Supplement: S38 Fig — Mean rate scalar was calculated from the mean of the rate scalars for the indicated group in the post-burn-in posterior distribution of the variable-rates evolutionary model estimated modelled using BayesTraits. Mean rates were scaled by dividing by the sum of the branch lengths in the corresponding time-scaled subtree. Models used the traditional Dinosauria phylogenetic topology (Saurischia and Ornithischia as sister clades). Mean rate scalars were compared between groups Modelled using nonparametric t tests; significantly different distributions are indicated with ****p < 0.00005. Data and code archived at www.github.com/rnfelice/Dinosaur_Skulls. (PDF) [file pbio.3000801.s038.pdf]

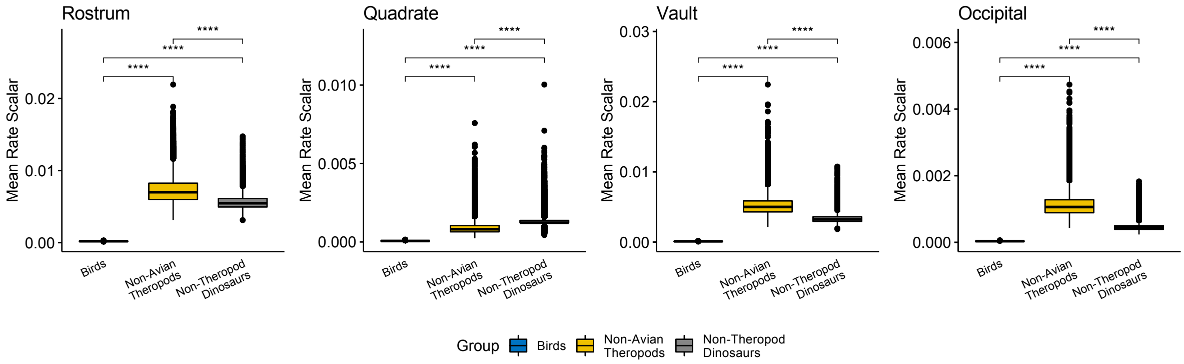

S38 Fig. Comparison of per-group evolutionary rate scalars with global Procrustes superimposition. Mean rate scalar was calculated from the mean of the rate scalars for the indicated group in the post-burn-in posterior distribution of the variable-rates evolutionary model estimated Modelled using BayesTraits. Mean rates were scaled by dividing by the sum of the branch lengths in the corresponding time-scaled subtree. Models used the traditional Dinosauria phylogenetic topology (Saurischia and Ornithischia as sister clades). Mean rate scalars were compared between groups Modelled using non-parametric t-tests; significantly different distributions are indicated with **** (p<0.00005). Data and code archived at www.github.com/rnfelice/Dinosaur_Skulls.
