# Supplementary material for: Decelerated dinosaur skull evolution with the origin of birds
Source: PLoS Biol. 2020 Aug 18;18(8):e3000801. doi: 10.1371/journal.pbio.3000801 (PMC7437466; doi:10.1371/journal.pbio.3000801)
Supplement: S40 Fig — Birds do not have the highest rates of evolution in any cranial region. Rate of evolution was calculated using the σmult metric [39]. Because overall sampling for birds was higher than for non-avian dinosaurs, we subsampled the birds to 1 species per order for 100 iterations. Rates were compared between groups using nonparametric t tests; significantly different distributions are indicated with ****p < 0.00005). Data and code archived at www.github.com/rnfelice/Dinosaur_Skulls. (PDF) [file pbio.3000801.s040.pdf]

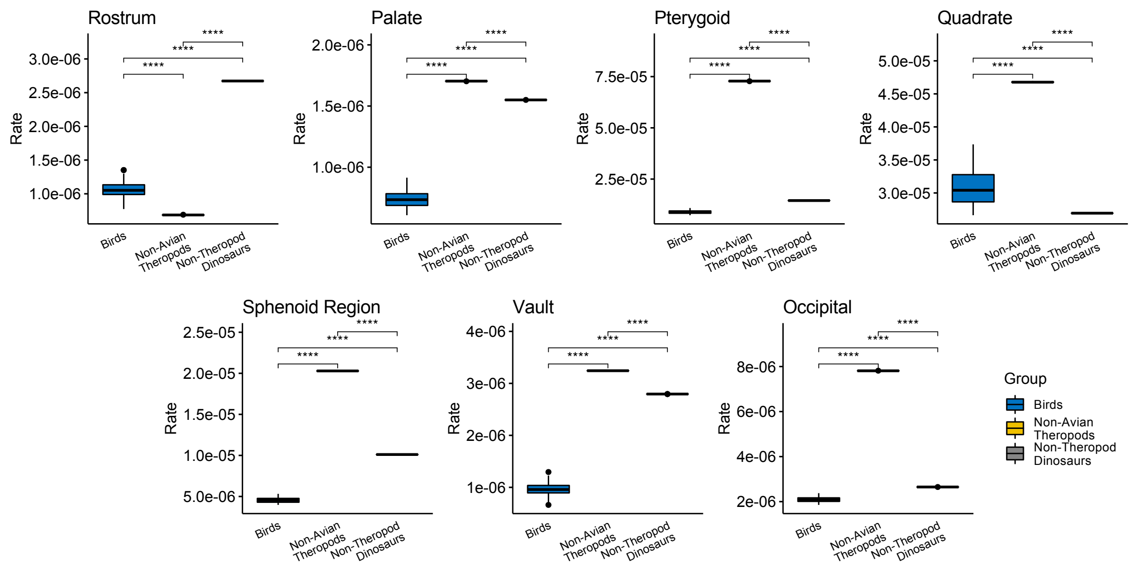

S40 Fig. Comparison of per-group evolutionary rates calculated using a phylogeny dated with the fossilized birth-death model. Birds do not have the highest rates of evolution in any cranial region. Rate of evolution was calculated using the σ_{mult} metric [39]. Because overall sampling for birds was higher than for non-avian dinosaurs, we subsampled the birds to 1 species per order for 100 iterations. Rates were compared between groups using non-parametric t-tests; significantly different distributions are indicated with **** ($p<0.00005$). Data and code archived at www.github.com/rnfelice/Dinosaur_Skulls.
