# Supplementary material for: Decelerated dinosaur skull evolution with the origin of birds
Source: PLoS Biol. 2020 Aug 18;18(8):e3000801. doi: 10.1371/journal.pbio.3000801 (PMC7437466; doi:10.1371/journal.pbio.3000801)
Supplement: S41 Fig — Rate of evolution was calculated using the σmult metric [39]. In addition to birds being subsampled to one species per order as in S38 Fig, one species was randomly removed from each dinosaur group in each of the 100 iterations. Rates were compared between groups using nonparametric t tests; significantly different distributions are indicated with ****p < 0.00005). Data and code archived at www.github.com/rnfelice/Dinosaur_Skulls. (PDF) [file pbio.3000801.s041.pdf]

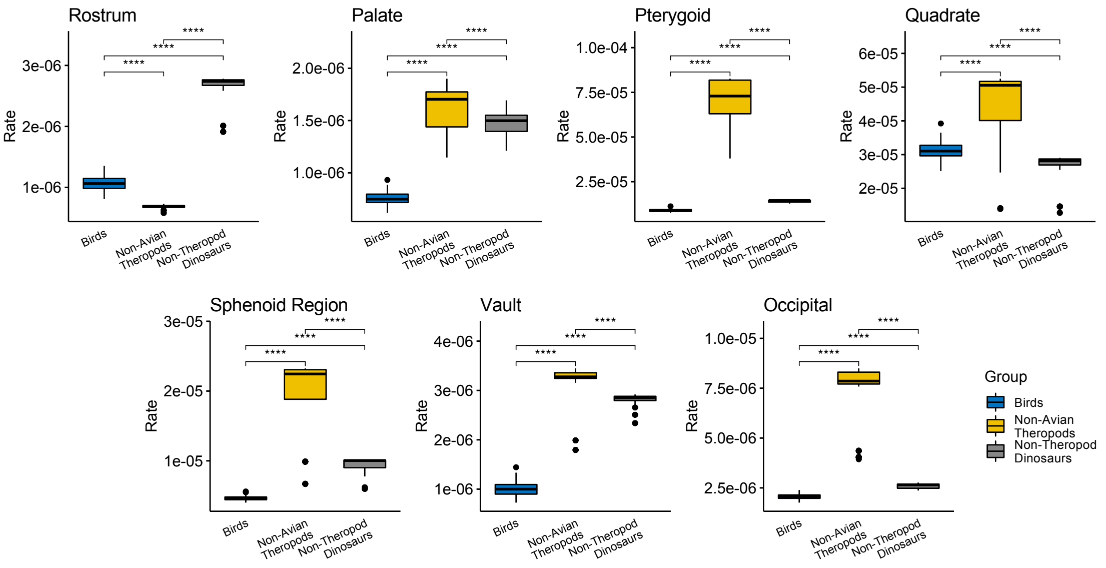

S41 Fig. Evaluating the effects of influential taxa on per-group evolutionary rates. Rate of evolution was calculated using the σ_{mult} metric [39]. In addition to birds being subsampled to one species per order as in Fig. S38, one species was randomly removed from each dinosaur group in each of the 100 iterations. Rates were compared between groups using non-parametric t-tests; significantly different distributions are indicated with **** (p<0.00005). Data and code archived at www.github.com/rnfelice/Dinosaur_Skulls.
