# Supplementary material for: Decelerated dinosaur skull evolution with the origin of birds
Source: PLoS Biol. 2020 Aug 18;18(8):e3000801. doi: 10.1371/journal.pbio.3000801 (PMC7437466; doi:10.1371/journal.pbio.3000801)
Supplement: S42 Fig — Rates calculated using the FBD-dated phylogenetic hypothesis. The high rates of evolution in the non-avian dinosaur quadrate observed in Fig 2 are likely a result of changes in the relative position of the jaw joint relative to the rest of the skull. The results presented in S37 Fig utilise separate Procrustes superimpositions for each region, and thus do not preserve differences in position among skull region. Here, under global Procrustes imposition, non-avian dinosaurs have significantly higher rates of quadrate evolution compared to birds. Rates were compared between groups using nonparametric t tests; significantly different distributions are indicated with ****p < 0.00005. Data and code archived at www.github.com/rnfelice/Dinosaur_Skulls. (PDF) [file pbio.3000801.s042.pdf]

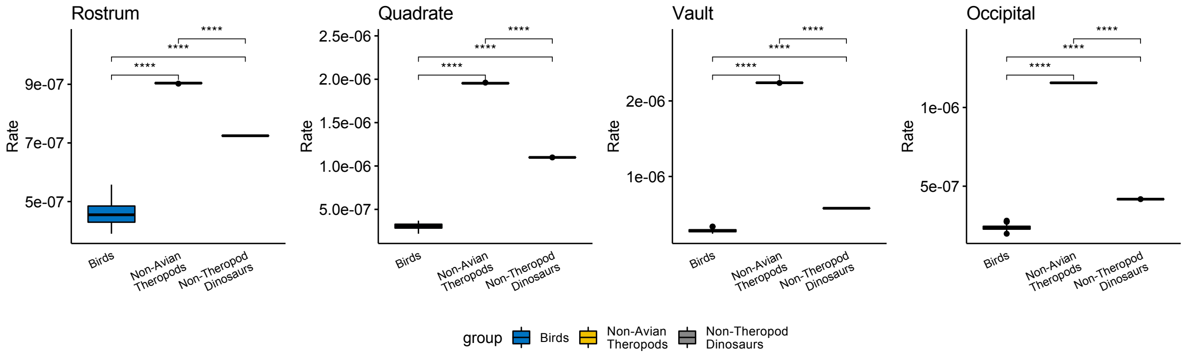

superimposition. Rates calculated using the FBD dated phylogenetic hypothesis. The high rates of evolution in the non-avian dinosaur quadrate observed in Fig. 2 are likely a result of changes in the relative position of the jaw joint relative to the rest of the skull. The results presented in Fig. S37 utilize separate Procrustes superimpositions for each region, and thus do not preserve differences in position among skull region. Here, under global Procrustes imposition, non-avian dinosaurs have significantly higher rates of quadrate evolution compared to birds. Rates were compared between groups using non-parametric t-tests; significantly different distributions are indicated with **** ($p<0.00005$). Data and code archived at www.github.com/rnfelice/Dinosaur_Skulls.
