# Supplementary material for: Decelerated dinosaur skull evolution with the origin of birds
Source: PLoS Biol. 2020 Aug 18;18(8):e3000801. doi: 10.1371/journal.pbio.3000801 (PMC7437466; doi:10.1371/journal.pbio.3000801)
Supplement: S43 Fig — Bivariate plots of disparity (Procrustes variance), mean evolutionary rate, and within-partition trait correlation (integration). Values were calculated for the 9-module dataset (A–C) and the 11-module dataset (D–F). Disparity is corrected by the number of landmarks and sliding semi-landmarks in the region. Within-module correlation values derived from ref [33]. Rates calculated using the traditional dinosaur phylogenetic topology, dated with the MBL method. Data and code archived at www.github.com/rnfelice/Dinosaur_Skulls. (PDF) [file pbio.3000801.s043.pdf]

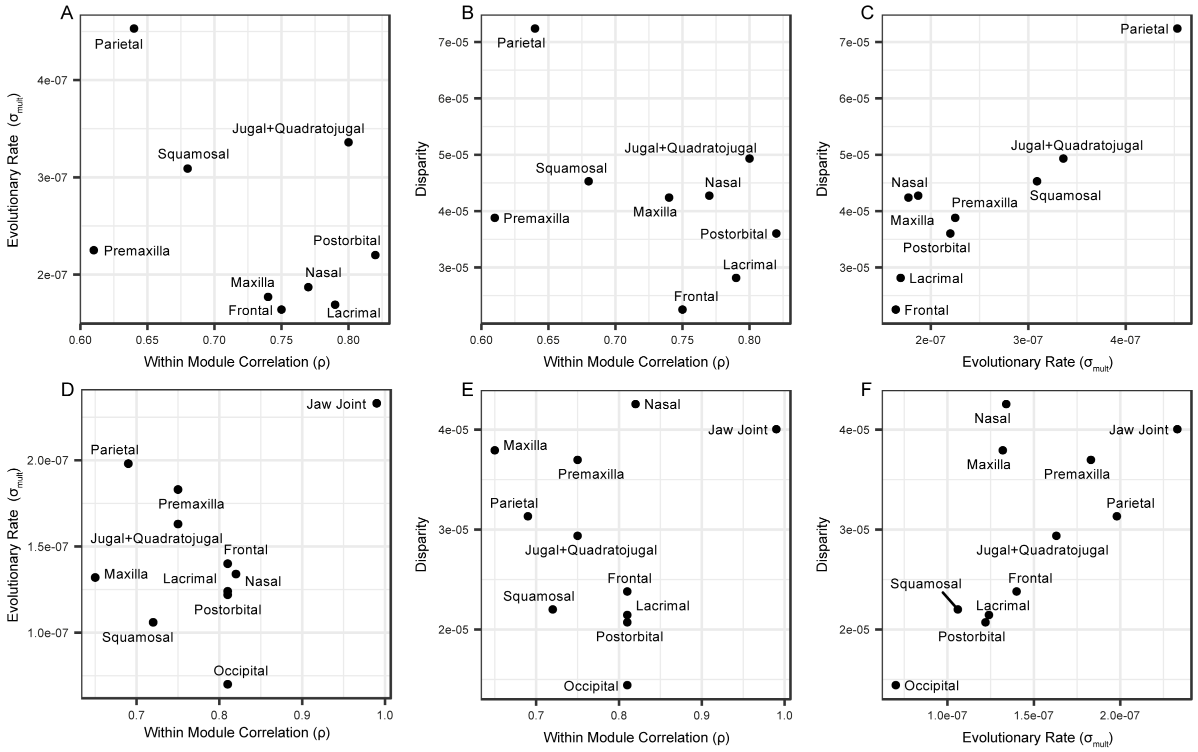


**S43 Fig. Rate, Disparity and Integration**.Bivariate plots of disparity (Procrustes variance), mean evolutionary rate, and within-partition trait correlation (integration). Values were calculated for the 9-module dataset(A-C) and the 11-module dataset (D-F). Disparity is corrected by the number of landmarks and sliding semi-landmarks in the region. Within-module correlation values derived from ref [33]. Rates calculated using the traditional dinosaur phylogenetic topology, dated with the minimum branch lengths method. Data and code archived at www.github.com/rnfelice/Dinosaur_Skulls.
