# Supplementary material for: Decelerated dinosaur skull evolution with the origin of birds
Source: PLoS Biol. 2020 Aug 18;18(8):e3000801. doi: 10.1371/journal.pbio.3000801 (PMC7437466; doi:10.1371/journal.pbio.3000801)
Supplement: S44 Fig — Calculated using the morphol.disparity function in the geomorph R package. Each cranial region except the quadrate is less disparate in birds than in non-avian groups. Data and code archived at www.github.com/rnfelice/Dinosaur_Skulls. (PDF) [file pbio.3000801.s044.pdf]

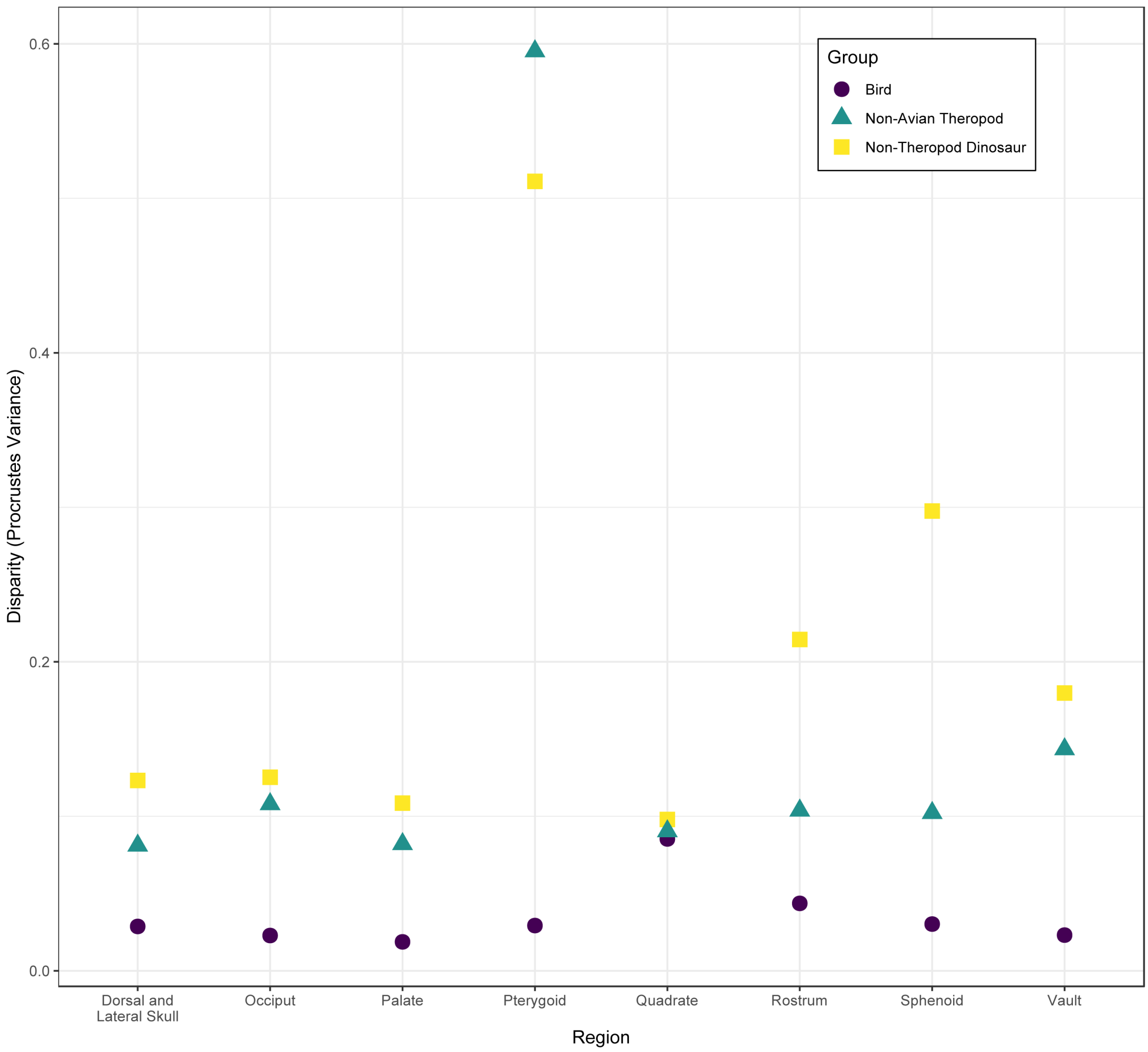

S44 Fig. Phenotypic Disparity Across Groups. Calculated using the morphol.disparity function in the geomorph R package. Each cranial region except the quadrate is less disparate in birds than in non-avian groups. Data and code archived at www.github.com/rnfelice/Dinosaur_Skulls.
