# Supplementary material for: Decelerated dinosaur skull evolution with the origin of birds
Source: PLoS Biol. 2020 Aug 18;18(8):e3000801. doi: 10.1371/journal.pbio.3000801 (PMC7437466; doi:10.1371/journal.pbio.3000801)
Supplement: S45 Fig — The extant bird portion of the phylogeny has shorter branch lengths due to denser sampling, but this effect is removed by the subsampling procedure. Data and code archived at www.github.com/rnfelice/Dinosaur_Skulls. (PDF) [file pbio.3000801.s045.pdf]

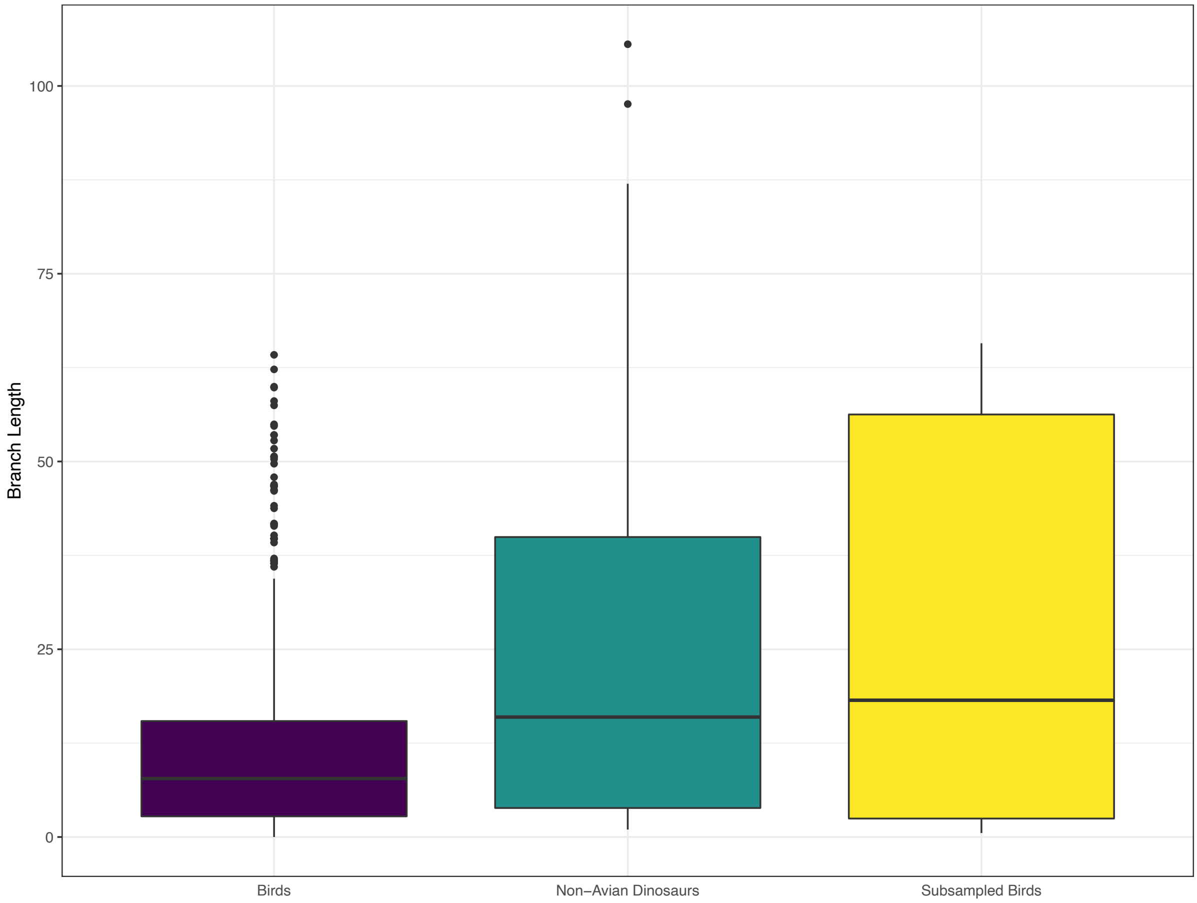

S45 Fig. Comparison of branch lengths among groups. The extant bird portion of the phylogeny has shorter branch lengths due to denser sampling, but this effect is removed by the subsampling procedure. Data and code archived at www.github.com/rnfelice/Dinosaur_Skulls.
