# Supplementary material for: Decelerated dinosaur skull evolution with the origin of birds
Source: PLoS Biol. 2020 Aug 18;18(8):e3000801. doi: 10.1371/journal.pbio.3000801 (PMC7437466; doi:10.1371/journal.pbio.3000801)
Supplement: S46 Fig — Linear regression reveals that the relationship between rate and branch length is not significant (R2 = 0.003, p = 0.07). Data and code archived at www.github.com/rnfelice/Dinosaur_Skulls. (PDF) [file pbio.3000801.s046.pdf]

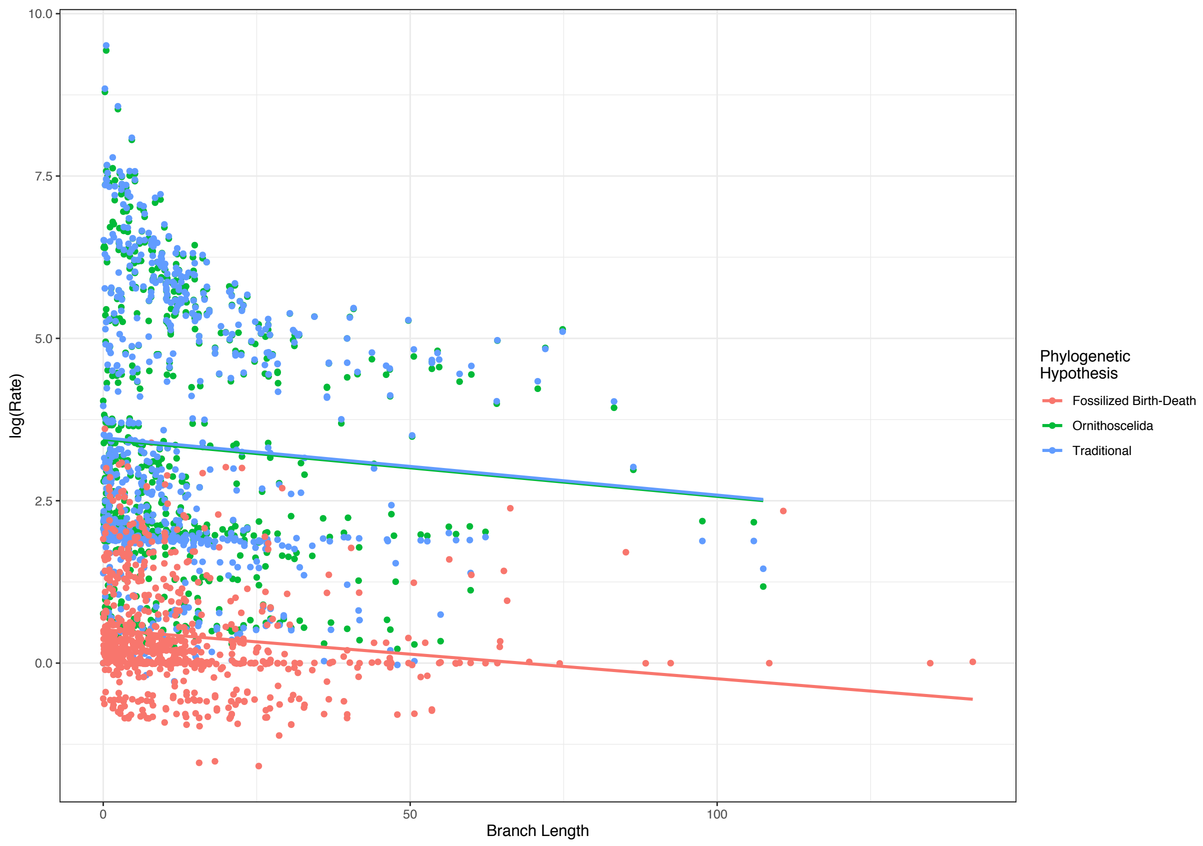

S46 Fig. The relationship between branch length and log-transformed rate. Linear regression reveals that the relationship between rate and branch length is not significant (R^2 = 0.003, p = 0.07). Data and code archived at www.github.com/rnfelice/Dinosaur_Skulls.
