# Supplementary material for: Decelerated dinosaur skull evolution with the origin of birds
Source: PLoS Biol. 2020 Aug 18;18(8):e3000801. doi: 10.1371/journal.pbio.3000801 (PMC7437466; doi:10.1371/journal.pbio.3000801)
Supplement: S47 Fig — The first 4 principal components axes explain a cumulative 66% of the total variance. Grey silhouettes represent the reconstructed shape at the corresponding region of morphospace. Birds and non-avian dinosaurs largely occupy distinct regions of morphospace. Diplodocus, with its elongate rostrum and posteriorly positioned nares, is convergent with birds with high PC axis 1 and moderate PC axis 2 scores. The domed skulls of pachycephalosaurs cause them to group with birds with high negative PC 1 scores Data and code archived at www.github.com/rnfelice/Dinosaur_Skulls. PC, principal component (PDF) [file pbio.3000801.s047.pdf]

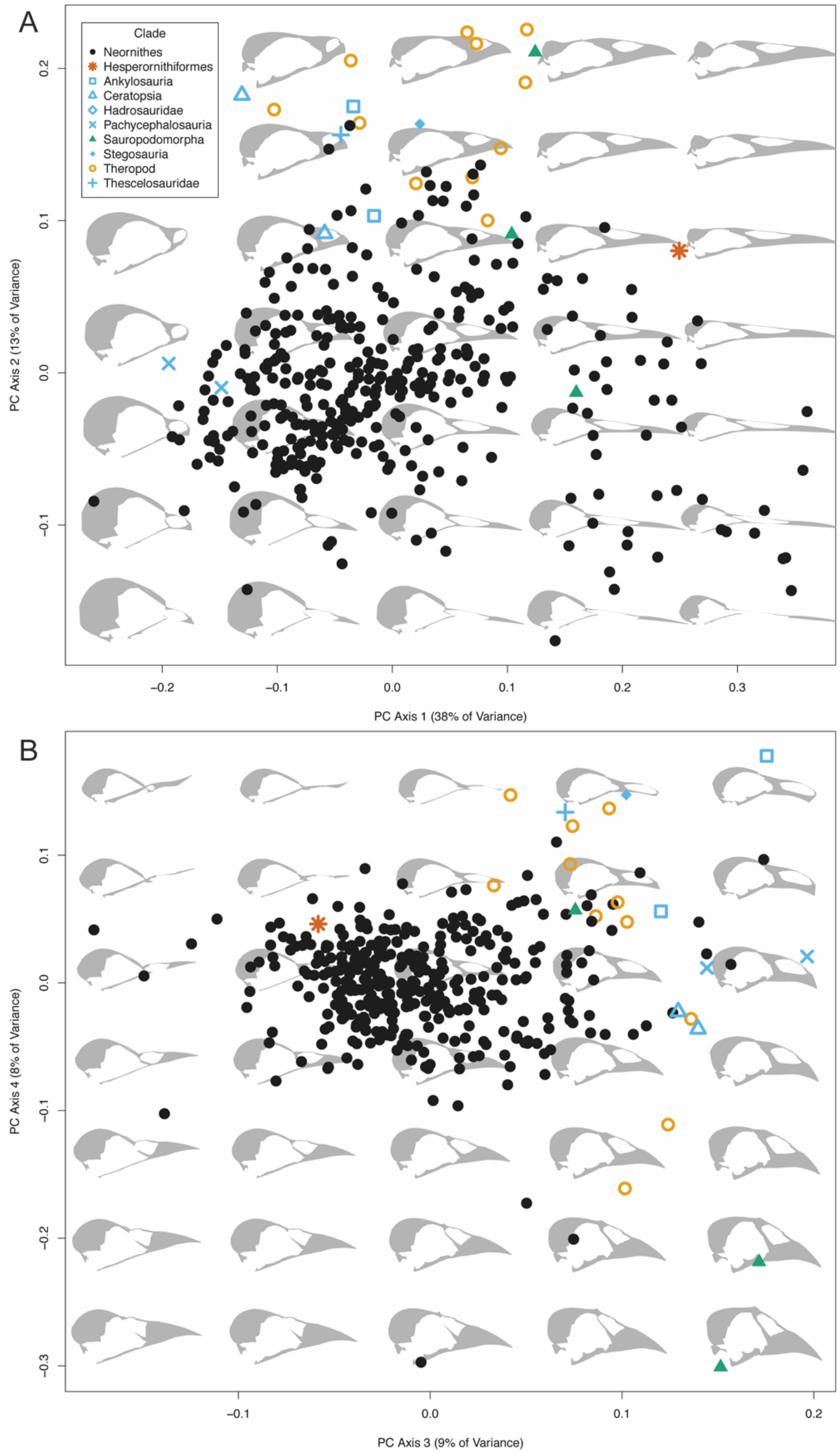

S47 Fig. Cranial morphospace for the rostrum, vault, and occipital regions. The first four principal components axes explain a cumulative 66% of the total variance. Grey silhouettes represent the reconstructed shape at the corresponding region of morphospace. Birds and non-avian dinosaurs largely occupy distinct regions of morphospace. Diplodocus, with its elongate rostrum and posteriorly positioned nares, is convergent with birds with high PC axis 1 and moderate PC axis 2 scores. The domed skulls of pachycephalosaurs cause them to group with birds with high negative PC 1 scores Data and code archived at www.github.com/rnfelice/Dinosaur_Skulls.
