# Supplementary material for: Decelerated dinosaur skull evolution with the origin of birds
Source: PLoS Biol. 2020 Aug 18;18(8):e3000801. doi: 10.1371/journal.pbio.3000801 (PMC7437466; doi:10.1371/journal.pbio.3000801)
Supplement: S1 Table — Calculated with the full dataset using the traditional dinosaur phylogenetic topology dated with the MBL method. Shaded cells indicate significantly faster rate than birds. (PDF) [file pbio.3000801.s048.pdf]

Region	Rate		
	Bird	Non-Avian Theropod	Non-Theropod Dinosaur
**Rostrum**	1.70E-06	1.13E-06	4.93E-06
**Occiput**	5.57E-06	1.62E-05	4.38E-06
**Vault**	1.88E-06	6.77E-06	5.07E-06
**Palate**	1.54E-06	2.75E-06	2.40E-06
**Pterygoid**	1.82E-05	1.46E-04	2.57E-05
**Quadrate**	1.35E-04	9.57E-05	3.32E-05
**Pterygoid+Quadrate**	5.44E-06	1.39E-05	9.51E-06
**Sphenoid**	8.88E-06	4.19E-05	1.01E-05

S1 Table. Evolutionary rates for each cranial region compared across groups. Calculated with the full dataset using the traditional dinosaur phylogenetic topology dated with the minimum branch lengths method. Shaded cells indicate significantly faster rate than birds.
