# Supplementary material for: Decelerated dinosaur skull evolution with the origin of birds
Source: PLoS Biol. 2020 Aug 18;18(8):e3000801. doi: 10.1371/journal.pbio.3000801 (PMC7437466; doi:10.1371/journal.pbio.3000801)
Supplement: S2 Table — Calculated using the morphol.disparity function in the geomorph R package. (PDF) [file pbio.3000801.s049.pdf]

Bird	Non-Theropod Dinosaur	Non-Avian Theropod	Region	Number of Surface Landmarks
0.044	0.214	0.104	Rostrum	244
0.023	0.125	0.108	Occiput	63
0.023	0.180	0.143	Vault	138
0.019	0.109	0.082	Palate	138
0.029	0.511	0.595	Pterygoid	30
0.085	0.098	0.090	Quadrate	15
0.030	0.298	0.102	Sphenoid	50
0.029	0.123	0.081	Dorsal and Lateral Skull	445

S2 Table. Phenotypic disparity of each cranial region. Calculated using the morphol.disparity function in the geomorph R package.
