# Supplementary material for: Decelerated dinosaur skull evolution with the origin of birds
Source: PLoS Biol. 2020 Aug 18;18(8):e3000801. doi: 10.1371/journal.pbio.3000801 (PMC7437466; doi:10.1371/journal.pbio.3000801)
Supplement: S3 Table — Calculated using the morphol.disparity function in the geomorph R package. (PDF) [file pbio.3000801.s050.pdf]

Region	Group	Bird	Non-Theropod Dinosaur
Rostrum	Non-Theropod Dinosaur	0.001	
	Non-Avian Theropod	0.007	0.001
Occiput	Non-Theropod Dinosaur	0.001	
	Non-Avian Theropod	0.001	0.11
Vault	Non-Theropod Dinosaur	0.001	
	Non-Avian Theropod	0.001	0.053
Palate	Non-Theropod Dinosaur	0.001	
	Non-Avian Theropod	0.001	0.041
Pterygoid	Non-Theropod Dinosaur	0.001	
	Non-Avian Theropod	0.001	0.162
Quadrate	Non-Theropod Dinosaur	0.532	
	Non-Avian Theropod	0.824	0.787
Jaw Joint	Non-Theropod Dinosaur	0.001	
	Non-Avian Theropod	0.001	0.195
Sphenoid	Non-Theropod Dinosaur	0.001	
	Non-Avian Theropod	0.004	0.001
Dorsal and Lateral Skull	Non-Theropod Dinosaur	0.001	
	Non-Avian Theropod	0.001	0.004

S3 Table. Significance values for pairwise comparisons of disparity. Calculated using the morphol.disparity function in the geomorph R package
