# Supplementary material for: Decelerated dinosaur skull evolution with the origin of birds
Source: PLoS Biol. 2020 Aug 18;18(8):e3000801. doi: 10.1371/journal.pbio.3000801 (PMC7437466; doi:10.1371/journal.pbio.3000801)
Supplement: S5 Table — Anatomical descriptions of landmark locations. (DOCX) [file pbio.3000801.s052.docx]

Supplementary Data File 3:

Landmark Definitions for Non-avian Dinosaur Datasets

Bird specimens were landmarked following Felice and Goswami (2018)

Landmark definitions: Non-avian dinosaurs

| Number | Left side counterpart | Description |
| --- | --- | --- |
| 1 | 48 | Posterolateral point on maxilla |
| 2 | 49 | Posterolateral most point on maxilla, ventral side |
| 3 | 50 | Posterolateral most point on the premaxilla |
| 4 | NA | Anteromedial most point on the premaxilla (or rostral bone) |
| 5 | NA | Posterior midline point on dorsal surface of the maxilla |
| 6 | NA | Anterior midline point on nasal |
| 7 | 51 | Anterior most point on medial premaxillary part of the nasal |
| 8 | 52 | Posterodorsal most point on the maxillary part of the premaxilla |
| 9 | 53 | Dorsal most point on the ascending process of the maxilla |
| 10 | 54 | Ventral most point on the descending anterior ramus of the nasal |
| 11 | NA | Posterior midline point of the nasal (frontonasal contact) |
| 12 | NA | Posterior midline of frontal |
| 13 | 55 | Posterolateral most point on the frontal |
| 14 | 56 | Anterolateral most point on the frontal |
| 15 | NA | Posterior midline point on parietal |
| 16 | 57 | Dorsal midline point on supraoccipital |
| 17 | 58 | Dorsal midline of foramen magnum |
| 18 | 59 | Medial point on the exoccipital contact with occipital condyle |
| 19 | 60 | Lateral most point on exoccipital |
| 20 | 61 | Lateral most point of the exoccipital-squamosal suture |
| 21 | 62 | Dorsal midline point on the occipital condyle |
| 22 | NA | Ventral midline point on the occipital condyle |
| 23 | NA | Lateral contact on exoccipital-basioccipital suture |
| 24 | 63 | Midline point on basioccipital-basisphenoid suture |
| 25 | 64 | Anterolateral most point on the post orbital |
| 26 | 65 | Tip of the descending part of the postorbital |
| 27 | 66 | Posterior most point on the postorbital |
| 28 | 67 | On the jugal, the posterior lacrimal-jugal contact |
| 29 | 68 | Posterior most point on the postorbital process of the jugal |
| 30 | 69 | Anterior contact of the jugal or quadratojugal to maxilla |
| 31 | 70 | Posteroventral most point on the quadratojugal |
| 32 | 71 | Dorsal most point on the posterior part of the quadratojugal |
| 33 | 72 | Ventral most point on the squamosal |
| 34 | 73 | Anterolateral most point on the squamosal |
| 35 | 74 | Anteromedial most point on the squamosal (squamosal-parietal contact) |
| 36 | 75 | Lateral most point on jaw joint |
| 37 | 76 | Medial most point on the jaw joint |
| 38 | 77 | Posterior most point on the jaw joint |
| 39 | 78 | Anterodorsal most point on the jugal |
| 40 | 79 | Posterolateral most point on the prefrontal |
| 41 | 80 | Dorsolateral most point on the lacrimal |
| 42 | 81 | Anterior most point on the ventral extent of the lacrimal |
| 43 | 82 | Posteromedial point on the prefrontal |
| 44 | 83 | Posterolateral point on the nasal |
| 45 | 84 | Parietal fenestra-anterior point |
| 46 | 85 | Parietal fenestra-posteromedial point |
| 47 | 86 | Parietal fenestra-posterolateral point |

Semilandmark Curve Definitions: Non-avian Dinosaurs

| Curve Number | Description | Number of Semilandmarks |
| --- | --- | --- |
| 1 | Medial margin of the ventral surface of the premaxilla | 20 |
| 2 | Posterior margin of the ventral surface of the premaxilla | 20 |
| 3 | Lateral margin of the ventral surface of the premaxilla | 20 |
| 4 | Medial margin of the ventral surface of the maxilla | 20 |
| 5 | Posterior margin of the ventral surface of the maxilla | 20 |
| 6 | Lateral margin of the ventral surface of the maxilla | 20 |
| 7 | Anterior margin of the ventral surface of the maxilla | 20 |
| 8 | Lateral margin of the maxilla | 20 |
| 9 | Anterior margin of maxilla | 20 |
| 10 | Posterior margin of maxilla | 20 |
| 11 | Lateral margin of premaxilla | 20 |
| 12 | Midline of premaxilla | 20 |
| 13 | Narial margin of the premaxilla | 20 |
| 14 | Premaxilla-maxilla suture | 20 |
| 15 | Midline of nasal | 8 |
| 16 | Posterior margin of the nasal | 20 |
| 17 | Lateral margin of the nasal | 20 |
| 18 | Narial margin of the nasal | 8 |
| 19 | Premaxilla-nasal suture | 20 |
| 20 | Midline of frontal | 20 |
| 21 | Posterior margin of the frontal | 20 |
| 22 | Lateral margin of the frontal | 20 |
| 23 | Anterior margin of the frontal | 20 |
| 24 | Midline of parietal | 30 |
| 25 | Posterior margin of the parietal | 20 |
| 26 | Lateral margin of the parietal | 20 |
| 27 | Anterior margin of the parietal | 20 |
| 28 | Midline of the supraoccipital | 15 |
| 29 | Lateral side of foramen magnum | 20 |
| 30 | Ventral margin of the exoccipital | 15 |
| 31 | Dorsal margin of the exoccipital and supraoccipital | 20 |
| 32 | Lateral margin of the occipital condyle | 20 |
| 33 | Midline of occipital condyle | 20 |
| 34 | Lateral margin of the basioccipital | 20 |
| 35 | Midline of basioccipital | 20 |
| 36 | Posteromedial margin of basisphenoid | 20 |
| 37 | Lateral margin of basisphenoid | 20 |
| 38 | Midline of basisphenoid | 20 |
| 39 | Posteromedial margin of pterygoid | 20 |
| 40 | Lateral margin of pterygoid | 20 |
| 41 | Medial margin of the pterygoid | 20 |
| 42 | Medial margin of palatine | 30 |
| 43 | Posterior margin of pterygoid | 20 |
| 44 | Lateral margin of pterygoid | 20 |
| 45 | Anterior margin of pterygoid | 20 |
| 46 | Anterior margin of postorbital | 20 |
| 47 | Posteroventral margin of postorbital | 20 |
| 48 | Dorsomedial margin of postorbial | 20 |
| 49 | Anterodorsal margin of jugal | 20 |
| 50 | Jugal-maxilla contact | 20 |
| 51 | Ventral margin of jugal and quadratojugal | 20 |
| 52 | Posterior margin of quadratojugal | 20 |
| 53 | Ventral margin of inferior temporal fenestra (on the jugal and quadratojugal) | 20 |
| 54 | Superior margin of jugal | 20 |
| 55 | Anterior margin of squamosal along margin of inferior temporal fenestra | 20 |
| 56 | Anterodorsal margin of squamosal | 20 |
| 57 | Medial margin of the squamosal | 15 |
| 58 | Posterior margin of squamosal | 20 |
| 59 | Posterolateral margin of articular surface of quadrate | 20 |
| 60 | Posteromedial margin of articular surface of quadrate | 20 |
| 61 | Anterior margin of articular surface of quadrate | 20 |
| 62 | Posterior margin of the prefrontal and lacrimal | 20 |
| 63 | Anterior margin of the prefrontal and lacrimal | 20 |
| 64 | Anterolateral margin of parietal fenestra | 20 |
| 65 | Posterior margin of parietal fenestra | 20 |
| 66 | Anteromedial margin of parietal fenestra | 20 |
