# Supplementary material for: Decelerated dinosaur skull evolution with the origin of birds
Source: PLoS Biol. 2020 Aug 18;18(8):e3000801. doi: 10.1371/journal.pbio.3000801 (PMC7437466; doi:10.1371/journal.pbio.3000801)
Supplement: S6 Fig — Modelled using a variable-rates BM model of evolution. Data and code archived at www.github.com/rnfelice/Dinosaur_Skulls. (PDF) [file pbio.3000801.s006.pdf]

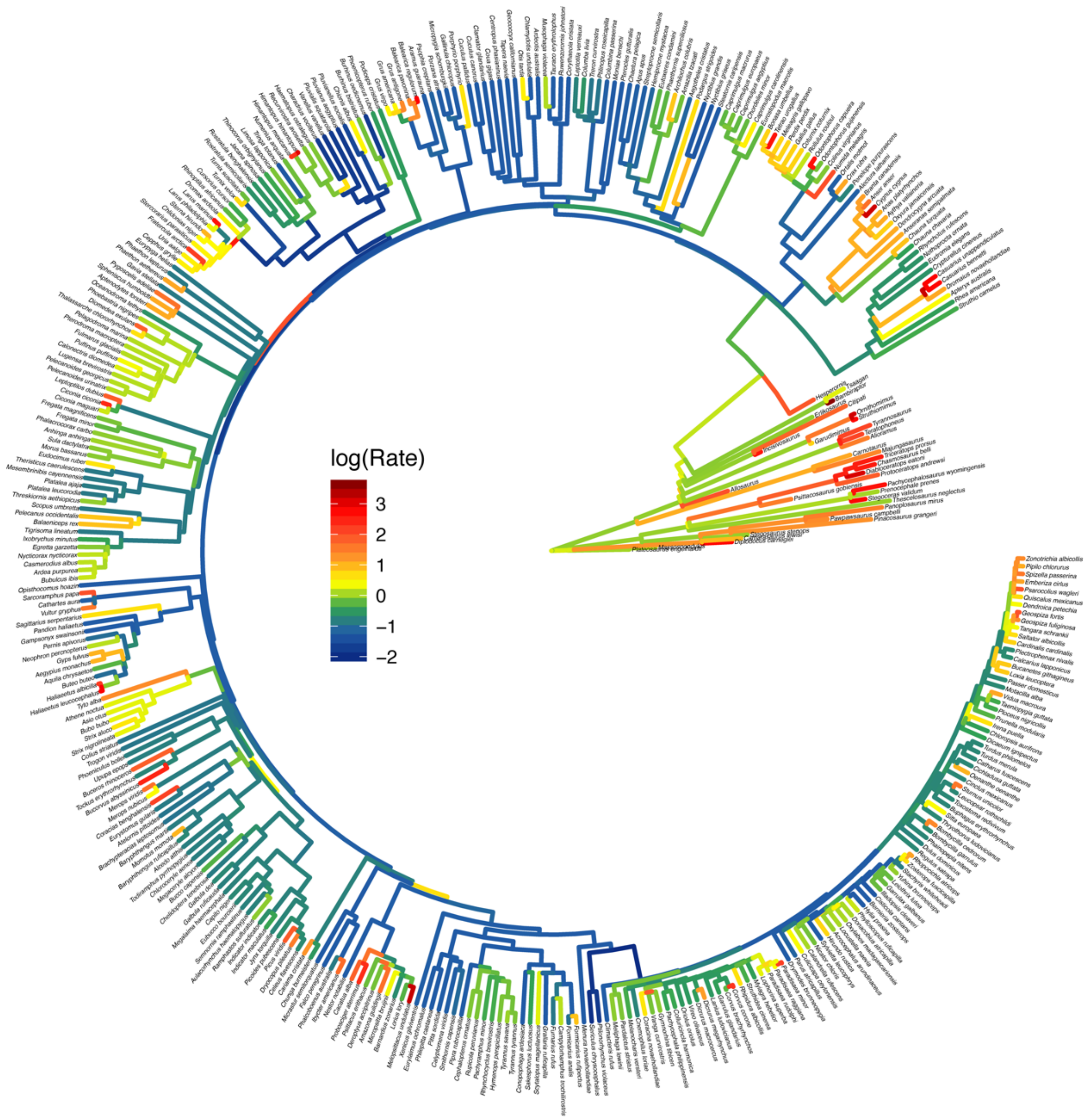

S6 Fig. Estimation of rates of cranial vault evolution (Ornithoscelida hypothesis). Modelled using a variable-rates Brownian motion model of evolution. Data and code archived at www.github.com/rnfelice/Dinosaur_Skulls.
